# Artificial intelligence in poultry processing: applications, validation gaps, and pathways toward intelligent and autonomous processing systems

**DOI:** 10.1016/j.psj.2026.107734

**Published:** 2026-09-07

**Authors:** Zahidul Hasan Tushar, Md Salman Mostafa

**Affiliations:** Department of Animal Science, Bangladesh Agricultural University, Mymensingh, 2202, Bangladesh

**Keywords:** Machine learning, Computer vision, Hyperspectral imaging, Food-safety monitoring, Meat quality

## Abstract

Artificial intelligence (AI) is emerging as a transformative tool for poultry processing by enabling rapid, nondestructive, and adaptive interpretation of images, spectra, sensor signals, and production records. This review critically examines AI applications across the poultry-processing chain, including live-bird receiving, slaughter, scalding, defeathering, evisceration, carcass inspection, chilling, antimicrobial control, cut-up, deboning, meat-quality assessment, breast-muscle abnormality detection, further processing, packaging, microbial monitoring, and predictive food-safety management. Particular attention is given to computer vision, machine learning, deep learning, near-infrared and hyperspectral imaging, electronic sensing, multimodal data fusion, robotics, and predictive modeling. Reported studies demonstrate strong potential for defect classification, carcass and portion localization, foreign-material detection, microbial-load estimation, freshness assessment, yield prediction, and process optimization. However, the literature establishes technical feasibility more convincingly than commercial reliability. Many models are developed from small, single-source datasets, use random data partitioning that may permit leakage, rely on uncertain reference labels, or report overall accuracy without class-specific performance, calibration, uncertainty, and external validation. Domain shifts caused by differences among flocks, plants, seasons, equipment, lighting, product orientation, and processing conditions remain major barriers to deployment. AI should therefore be implemented as part of an integrated sensor–model–decision–actuator system rather than as an isolated algorithm or replacement for validated process controls and human expertise. Future progress requires multi-plant datasets, prospective commercial-line testing, uncertainty-aware models, hygienic and low-latency hardware, economic assessment, transparent governance, and clearly defined human oversight. The transition from experimental prediction to intelligent and autonomous poultry processing will depend on whether AI can consistently improve safety, quality, yield, sustainability, and operational decision-making under realistic industrial conditions.

## Primary audience

Plant managers, Quality assurance personnel, Researchers, Complex managers

## Introduction

Poultry meat is an important source of high-quality animal protein because it is nutritious, affordable, versatile, widely accepted, and comparatively efficient to produce. Rising production and demand for portioned and further-processed products have encouraged centralized plants capable of processing approximately 15,000 birds per hour on a single line (Barbut and Leishman, 2022). At this scale, processors must simultaneously maintain food safety, product consistency, yield, regulatory compliance, worker safety, and environmental performance (O’Bryan et al., 2026). Although higher line speeds increase throughput, they reduce the time available for inspection, equipment adjustment, defect removal, and corrective action (Barbut, 2014).

Poultry carcasses also vary in size, anatomy, tissue distribution, and processing behavior because of genetics, age, sex, live weight, flock management, preslaughter conditions, and processing history. Conventional equipment performs most consistently when products are uniform and predictably positioned; therefore, settings developed for an average carcass may not accommodate the biological variation encountered commercially (Barbut and Leishman, 2022; Lyu et al., 2025). Breast-muscle abnormalities, including woody breast (WB), white striping (WS), and spaghetti meat (SM), further alter appearance, composition, texture, water-holding capacity, and functionality (Baldi et al., 2018; Petracci et al., 2019; Tushar et al., 2026). WB is associated with abnormal hardness and impaired processing properties, whereas SM involves loss of structural integrity and separation of muscle-fiber bundles (Barbut et al., 2024; Petracci et al., 2019). These abnormalities complicate grading, portioning, formulation, and value recovery (Barbut and Leishman, 2022).

Processing plants must additionally detect contamination, incomplete processing, carcass defects, foreign materials, misclassification, package failures, and quality deviations. Many reference methods for microbial load, composition, freshness, and technological quality are destructive, labor-intensive, or too slow for real-time decisions (Shi et al., 2021). Although Fourier-transform infrared spectroscopy and multispectral imaging combined with multivariate or machine-learning models have shown promise for rapid spoilage assessment, performance differences between calibration datasets and independent batches indicate that strong internal results do not necessarily demonstrate industrial robustness (Spyrelli et al., 2021a; Spyrelli et al., 2021b).

Modern poultry plants rely on mechanization and automation for slaughter, evisceration, chilling, cut-up, deboning, further processing, and packaging. These systems provide high throughput and repeatability but generally operate through predetermined movements, thresholds, and rules. Their performance can decline when carcass size, anatomy, orientation, illumination, or operating conditions vary beyond predefined limits (Barbut, 2014; Lyu et al., 2025). Such variability is particularly challenging in poultry processing because deformable tissues, slippery surfaces, occlusion, rapid line movement, and strict sanitation requirements complicate sensing, cutting, and robotic handling (Lyu et al., 2025; O’Bryan et al., 2026).

Artificial intelligence (AI)-enabled systems offer a more adaptive approach by interpreting images, spectra, sensor measurements, and production records to support inspection, classification, quality prediction, cutting guidance, process control, and robotic operations (Barbar et al., 2022; Jia et al., 2026). In poultry processing, AI has been combined with RGB, depth, thermal, multispectral, and hyperspectral imaging; near-infrared and Fourier-transform infrared spectroscopy; electronic sensors; and production data. Reported applications include carcass inspection, breast-myopathy classification, freshness and microbial-quality estimation, foreign-material detection, yield prediction, robotic handling, and operational optimization (Jia et al., 2026; Shi et al., 2021). However, AI should be regarded as part of an integrated processing system rather than a replacement for appropriate equipment design, reliable sensing, sanitation, maintenance, and human expertise (Ahlin, 2022; Lyu et al., 2025).

The practical value of these technologies depends on more than high experimental accuracy. Systems must generate reliable predictions at commercially relevant speeds and under realistic plant conditions. For example, semisupervised deep learning with near-infrared hyperspectral imaging has demonstrated strong performance for detecting foreign materials on poultry meat, while model compression and hardware acceleration have improved inference speed (Campos et al., 2023; Khan et al., 2025). Nevertheless, much of this evidence remains based on laboratory studies or simulated processing conditions rather than long-term commercial-line validation. Human–AI collaboration may therefore be especially important, with automated systems conducting continuous screening while trained personnel review uncertain or high-risk cases (O’Bryan et al., 2026).

Model performance can be affected by dataset representativeness, class imbalance, reference-label uncertainty, sensor configuration, and variation among flocks, plants, seasons, and genetic lines (Lones, 2024). High overall accuracy may conceal poor detection of uncommon but operationally important defects, making class-specific sensitivity, specificity, precision, recall, calibration, and external-validation performance essential for meaningful evaluation (Saito and Rehmsmeier, 2015; Lones, 2024). Visual scoring, destructive laboratory assays, and microbiological reference methods also introduce uncertainty that should be reported transparently. Consequently, the key challenge is not simply demonstrating that an algorithm can identify a statistical pattern, but establishing that its predictions are reliable, actionable, economically justified, and stable under commercial processing conditions (Barbar et al., 2022; Lones, 2024).

Previous reviews have provided valuable perspectives on computer vision, spectroscopy, robotics, electronic sensing, machine learning, and related technologies in poultry, meat, and food-processing systems (Jia et al., 2026; Lyu et al., 2025; Shi et al., 2021). Collectively, these studies demonstrate the considerable potential of data-driven technologies for automated inspection, quality assessment, food-safety monitoring, and processing operations. As these technologies move closer to industrial application, however, an increasingly important consideration is not only whether a model performs well experimentally, but whether its performance remains reliable, actionable, and economically meaningful under commercial processing conditions (Ahlin, 2022; Lyu et al., 2025).

The present review therefore integrates AI applications across the poultry-processing chain while placing particular emphasis on the transition from experimental prediction to industrial implementation. In addition to technological applications, studies are critically considered in relation to dataset representativeness, reference-standard reliability, validation design, data leakage, class imbalance, uncertainty, external validation, domain shift, processing throughput, engineering integration, economic relevance, governance, and human oversight. The review further considers AI as part of an integrated sensor–model–decision–actuator system and examines whether predictive outputs can support practical interventions in processing, quality, safety, yield, and resource management. This combined processing-chain and implementation-oriented perspective is intended to provide researchers and industry stakeholders with a framework for evaluating both the capabilities and the readiness of AI technologies for poultry-processing applications.

Literature for this critical narrative review was identified through structured searches of Scopus, Web of Science, PubMed, and IEEE Xplore from database inception through July 2026. Search terms combined poultry-related terms (e.g., poultry, chicken, broiler, carcass, and poultry meat) with processing-related terms (e.g., slaughter, evisceration, chilling, deboning, inspection, quality, food safety, packaging, and robotics) and AI-related terms (e.g., artificial intelligence, machine learning, deep learning, computer vision, hyperspectral imaging, spectroscopy, neural networks, and predictive modeling). Reference lists of relevant reviews and primary studies were additionally screened. Studies were prioritized according to their relevance to poultry processing and were critically evaluated with particular attention to sample and processing context, validation approach, reported performance, and evidence of pilot- or commercial-scale deployment. The review was designed as a critical narrative synthesis rather than a formal systematic review or meta-analysis.

### AI foundations relevant to poultry processing

**Fig. 1:**
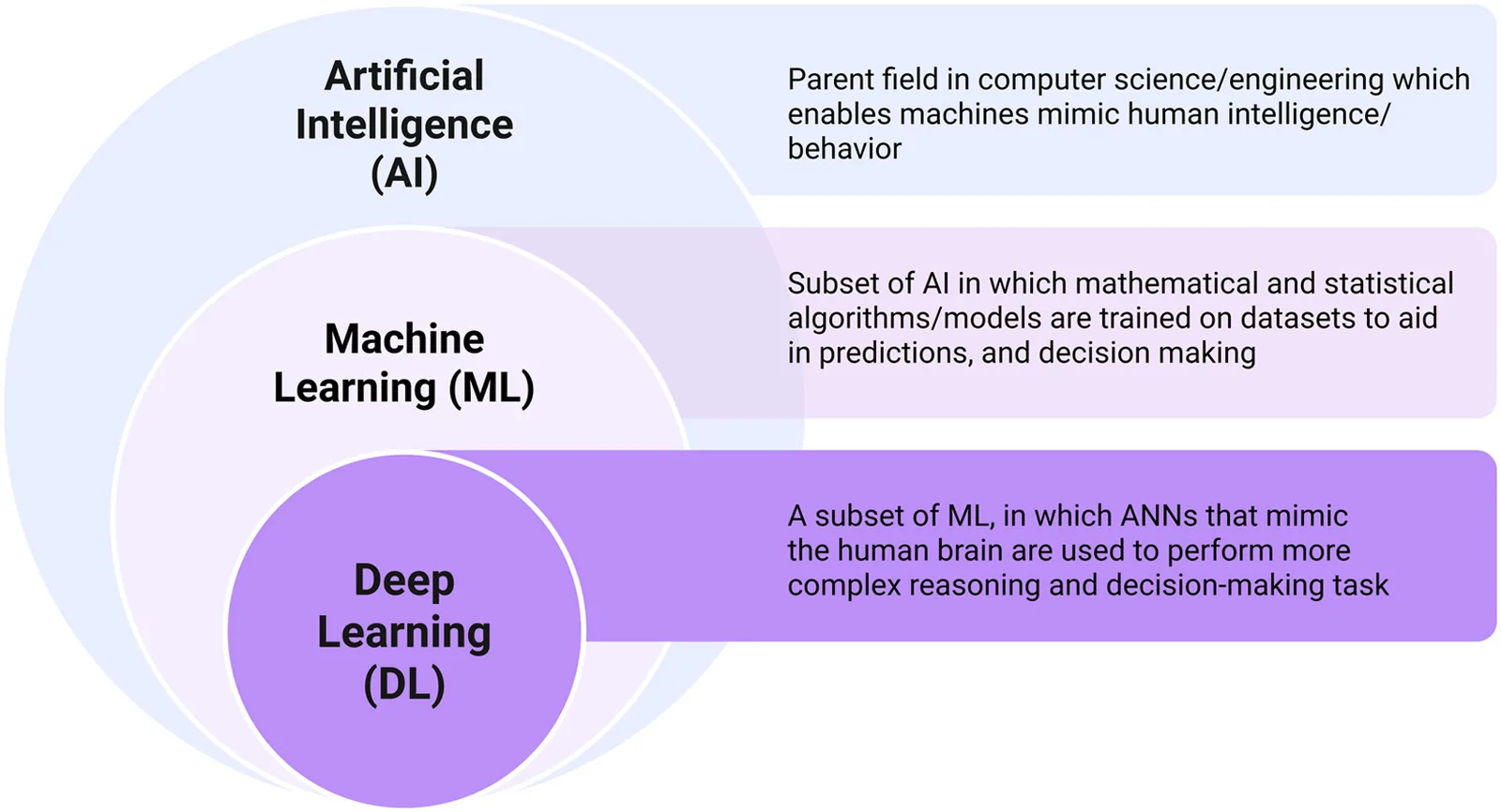
Relationship among artificial intelligence, machine learning, and deep learning. Created with BioRender.

***Artificial intelligence, machine learning, and deep learning.*** AI includes computational methods for perception, prediction, optimization, and decision-making. Machine learning (ML) is a subset of AI in which relationships are learned from data rather than specified entirely through fixed rules, while deep learning (DL) uses multilayer neural networks to learn representations from high-dimensional inputs such as images, spectra, and time-series data (Fig. 1) (Jordan and Mitchell, 2015; Lecun et al., 2015).

Not every automated or statistical system is AI-enabled. Equipment following a fixed cutting path or predetermined rejection threshold represents conventional automation, while principal component analysis and partial least-squares regression are generally considered chemometric methods. AI-enabled systems use learned relationships for classification, prediction, localization, optimization, or adaptive control (Lin et al., 2023).

Common poultry-processing applications include classification of normal and defective products, regression-based prediction of microbial load or quality attributes, and object detection or segmentation to locate contamination, foreign materials, anatomical structures, or defects. Spatial outputs are particularly valuable when predictions must guide trimming, cutting, robotic handling, or product rejection (Jia et al., 2026; Lyu et al., 2025).

***Learning strategies and model families.*** Supervised learning uses observations with known labels or measured outcomes. Common methods include logistic regression, support-vector machines, decision trees, random forests, gradient boosting, artificial neural networks, and convolutional neural networks (CNNs). Because reference values may come from visual scoring, microbiological testing, chemical analysis, or processing measurements, model reliability depends on both input quality and reference-measurement accuracy (Lin et al., 2023; Lones, 2024).

Unsupervised learning identifies patterns without predefined labels and may support clustering, dimensionality reduction, anomaly detection, or equipment monitoring. However, identified clusters require comparison with independent biological or operational information before they can be considered meaningful (Jordan and Mitchell, 2015).

Semi-supervised learning combines limited labeled data with larger quantities of unlabeled data, making it useful when expert annotation or destructive testing is expensive. Campos et al. (2023) for example, applied semi-supervised DL to hyperspectral detection of foreign materials on poultry meat. Transfer learning similarly reduces data requirements by adapting a pretrained model, although its effectiveness depends on similarity between the original and target domains (Pan and Yang, 2010).

Reinforcement learning selects actions according to feedback and could support process control, energy optimization, or robotic trajectory planning. However, unsafe or costly trial-and-error learning limits its direct use on commercial lines; simulation, digital twins, operational constraints, and supervisory safeguards are therefore needed.

CNNs are widely used for image classification, detection, and segmentation, whereas recurrent networks and transformers can model sequential data. No model is universally superior; selection should reflect data structure, sample size, interpretability, inference speed, and the consequences of prediction errors (Lecun et al., 2015; Lin et al., 2023).

***Sensors, data representations, and multimodal fusion*.** AI models interpret sensor-generated data rather than measuring poultry products directly. RGB cameras capture visible color, shape, surface defects, and orientation. Depth and three-dimensional imaging provide geometric information for anatomical localization, portioning, and robotic path planning, while thermal imaging captures surface-temperature patterns (Lin et al., 2023; Shi et al., 2021).

Near-infrared and Fourier-transform infrared spectroscopy provide molecular and compositional information, while multispectral and hyperspectral imaging combine spatial and spectral measurements. These technologies have been investigated for predicting composition, spoilage, contamination, foreign materials, and meat-quality attributes. Their limitations include large data volumes, calibration requirements, sensitivity to acquisition conditions, and imperfect relationships between surface measurements and internal product characteristics (Lin et al., 2023; Spyrelli et al., 2021a, 2021b).

Process data may include temperature, time, pressure, flow rate, antimicrobial concentration, motor load, vibration, production rate, and yield. Multimodal learning combines such measurements with images or spectra through feature-level, model-level, or prediction-level fusion (Baltrusaitis et al., 2019). Although complementary sensors may improve performance, poor synchronization, missing data, unequal spatial resolution, and calibration differences can increase error and complexity (Baltrusaitis et al., 2019; Hayes et al., 2023). Multimodal systems should therefore be compared with simpler single-sensor baselines to determine whether performance gains justify the added cost.

**Fig. 2:**
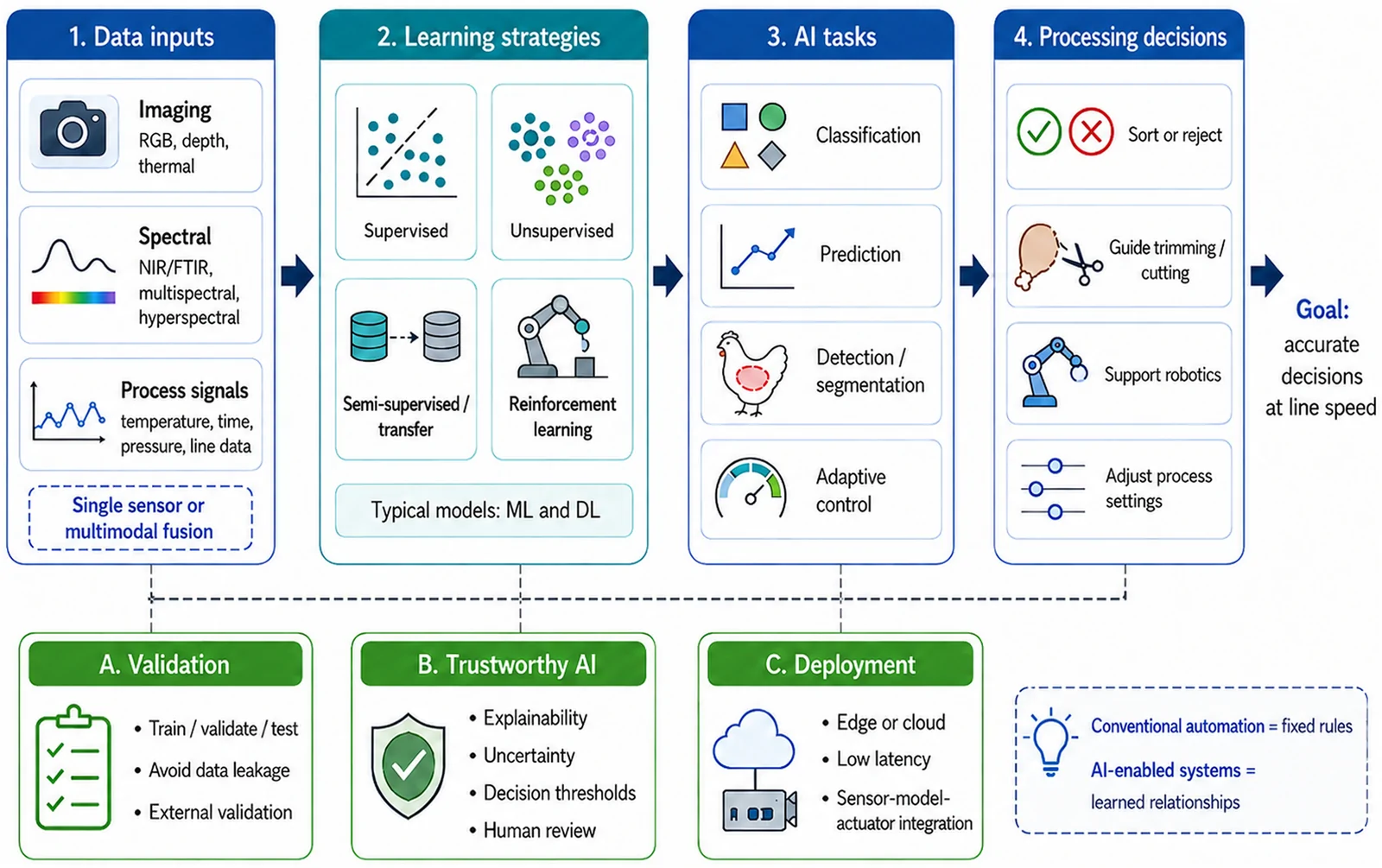
Conceptual workflow for developing and implementing AI-enabled systems in poultry processing.

***Model development and validation*.** An ML workflow generally includes data acquisition, reference measurement, preprocessing, data partitioning, model training, hyperparameter selection, validation, and final testing. Training data estimate model parameters, validation data support model selection, and an untouched test set provides the final performance assessment (Kapoor and Narayanan, 2023; Lones, 2024). The major stages involved in developing, validating, and implementing an AI-enabled poultry-processing system are summarized in Fig. 2.

Partitioning requires particular care because multiple images, spectra, or measurements may originate from the same carcass, flock, or processing day. Placing related observations in both training and testing datasets can cause data leakage and inflate performance. Data should therefore be separated at the highest relevant biological or operational level, such as carcass, flock, processing date, or plant (Kapoor and Narayanan, 2023).

Cross-validation estimates internal stability but does not replace external validation. Independent testing across production periods, flocks, plants, sensors, or operating conditions is needed because changes in lighting, carcass size, genetic line, temperature, line speed, and defect prevalence may cause domain shift and reduce performance (Lones, 2024).

Evaluation measures should match the task and consequences of error. Classification studies should report class-specific sensitivity, specificity, precision, recall, confusion matrices, and receiver-operating-characteristic or precision–recall measures. Precision–recall analysis is particularly useful when important defects are uncommon (Saito and Rehmsmeier, 2015). Regression studies should report prediction error, bias, coefficient of determination, and performance across the measurement range. Sample size, confidence intervals, throughput, and external-validation results are also necessary for meaningful interpretation.

***Explainability, uncertainty, and decision thresholds*.** Explainable AI methods attempt to identify the information influencing a prediction. Image heatmaps can highlight influential regions, while variable-importance methods can identify relevant wavelengths or processing parameters. These approaches may reveal whether a model relies on genuine product characteristics or unintended features such as backgrounds, labels, or lighting artifacts (Gunning et al., 2019).

Nevertheless, explanations do not prove that a model has learned a causal or biologically valid mechanism. Different methods may produce different interpretations, and plausible visual explanations can still be misleading. Explainability should therefore complement, not replace, independent validation.

Uncertainty is equally important because model predictions are not error-free. Neural-network probabilities may be poorly calibrated, especially under domain shift (Tomani et al., 2021). Calibration assesses whether predicted probabilities correspond with observed outcome frequencies and helps determine whether confidence estimates can support operational decisions.

Decision thresholds should reflect the consequences of errors. Food-safety systems may prioritize sensitivity to reduce false negatives, whereas excessive false positives can cause unnecessary product loss. A practical system may therefore process high-confidence predictions automatically while referring uncertain or high-risk cases for human review.

***Real-time inference and deployment*.** Commercial deployment requires the complete system to acquire data, preprocess inputs, generate predictions, communicate results, and activate an intervention within the available processing time. Latency, sensor coverage, product spacing, computing capacity, network reliability, and actuator response must therefore be assessed together.

Cloud computing provides substantial processing capacity but may introduce network dependence and communication delays. Edge computing performs analysis near the production equipment, reducing data transmission and response time (W. Shi et al., 2016). Compression, pruning, quantization, and hardware acceleration can further reduce computational demands. Khan et al. (2025) demonstrated faster inference for hyperspectral foreign-material detection through model compression and acceleration, although simulated throughput does not substitute for prospective commercial-line validation.

The appropriate unit of evaluation is consequently the integrated sensor–model–decision–actuator system. Even a highly accurate model has limited industrial value if it cannot operate at line speed, withstand sanitation, maintain calibration, integrate with existing equipment, or trigger a useful action. These principles provide the foundation for evaluating AI applications across poultry-processing operations.

***Framework for Evaluating AI Readiness in Poultry Processing.*** The practical value of AI in poultry processing cannot be determined from predictive performance alone; industrial usefulness also depends on whether predictions are reliable, timely, and connected to meaningful processing decisions and outcomes (Barbar et al., 2022; Lones, 2024). Accordingly, the applications discussed in this review are considered within an integrated sensor–model–decision–actuator framework. The sensor determines whether relevant biological or processing information can be acquired reliably; the model converts those measurements into a prediction, classification, or recommendation; the decision stage determines how the output should be interpreted and whether human review is required; and the actuator or operational response converts the prediction into a processing action, such as sorting, trimming, equipment adjustment, product diversion, or targeted inspection (Ahlin, 2022; Barbar et al., 2022; Lyu et al., 2025; O’Bryan et al., 2026).

Across these stages, technology readiness depends on several cross-cutting considerations: (1) data and reference quality, (2) model-development and validation rigor, (3) robustness to domain shift and production variability, (4) inference speed and engineering integration, and (5) operational, economic, regulatory, and human consequences of the resulting decision (Kapoor and Narayanan, 2023; Lones, 2024; Jia et al., 2026). Reliable deployment also requires appropriate characterization of predictive uncertainty, particularly when uncertain outputs may trigger consequential processing or food-safety decisions (Hüllermeier and Waegeman, 2021). This framework is used throughout the review to distinguish laboratory proof of concept from systems that have demonstrated meaningful readiness for commercial poultry-processing environments. High model accuracy should therefore be interpreted as one component of readiness rather than as evidence of successful industrial implementation (Lones, 2024; Lyu et al., 2025).

### AI applications in primary poultry processing and carcass inspection

**Fig. 3:**
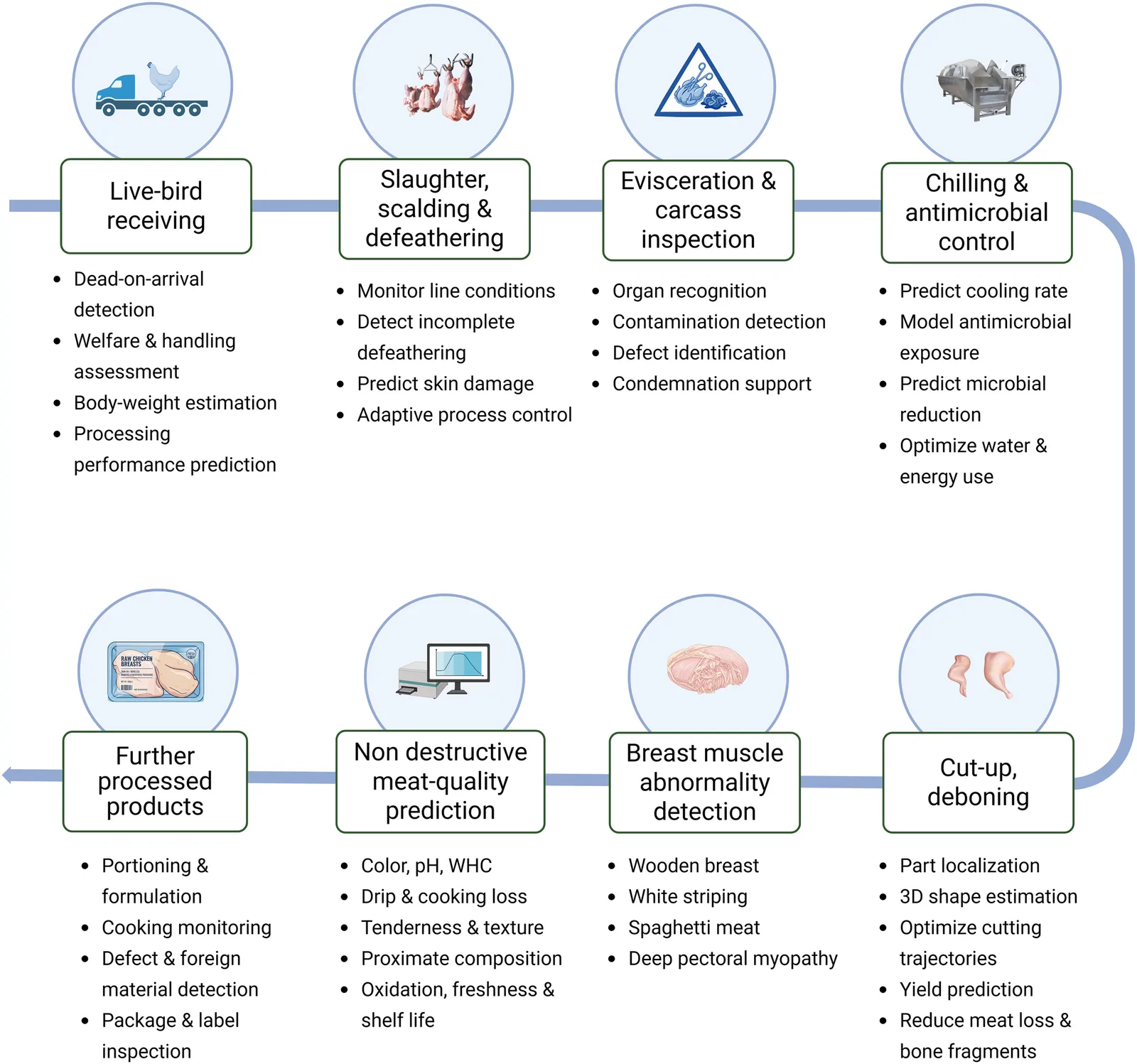
Representative applications of artificial intelligence across the poultry-processing chain. Created with BioRender.

Primary poultry processing extends from live-bird receiving through slaughter, defeathering, evisceration, carcass inspection, and sorting. Although these operations are highly mechanized, most machines still apply the same settings to carcasses that differ in size, shape, position, and condition. AI, particularly computer vision, can help processing systems respond to this biological variation. A practical way to view AI in this setting is as an additional set of eyes: it can continuously inspect products, recognize patterns, and provide information for sorting, equipment adjustment, or human review. However, the evidence is uneven. Spectral carcass-inspection systems have been tested continuously on commercial lines, whereas many recent deep-learning and robotic applications remain at the image-dataset, laboratory, or pilot stage (Lyu et al., 2025; Jia et al., 2026). Representative applications of AI across the poultry-processing chain, from live-bird receiving and primary processing to quality assessment, further processing, packaging, and food-safety monitoring, are summarized in Fig. 3. Representative AI applications spanning different stages of poultry processing and levels of validation and deployment readiness are summarized in Table 1. A more comprehensive evidence map, including AI/analytical approaches, sensing modalities, sample size and processing context, validation type, reported performance, and critical interpretation of deployment status, is provided in Supplementary Table S1.

**Table 1:** Representative AI applications in poultry processing with validation and deployment context.

Task	Modality	Sample size/plant context	Validation type	Reported metric	Deployment status	Key study
Stunning-state assessment	RGB/CMOS imaging; Faster-RCNN + residual module	2,319 original slaughter-line images augmented to 27,828; test set n = 5,568	Internal train/validation/test split after augmentation; single-line image dataset	Accuracy 98.06%; class sensitivities 98.41%, 98.20%, and 97.31%; 0.0822 s/image (∼43,700 birds/h)	Unknown / line-derived	Ye et al., 2020
On-line carcass-wholesomeness inspection	Line-scan VIS/NIR spectral imaging	Model development: 5,549 wholesome + 93 unwholesome carcasses; >100,000 carcasses inspected on a 140-bird/min commercial line	Continuous in-plant commercial-line operation	>99% wholesome and >96% unwholesome carcasses correctly classified	Commercial-line	Yang et al., 2008; Chao et al., 2011
Carcass-defect detection and grading	RGB imaging; transformer-based detection/segmentation (CarcassFormer)	7,321 images from a university pilot processing plant; 4,279 single-carcass and 3,042 multiple-carcass images; test set n = 900	Internal train/validation/test split; single pilot plant	For multiple-carcass images (ResNet-50): detection AP 90.45, defect AP 87.49, segmentation AP 98.96; no external-plant test	Pilot	Tran et al., 2024
Robotic evisceration and visceral localization	RGB machine vision + robotic manipulator/end-effector	Experimental evisceration system tested on chickens of two weight ranges; external plant-scale validation not reported	Prototype/experimental system evaluation	Maximum localization deviation 5 pixels (carcass) and 4 pixels (incision); 954 ms image processing; viscera residual 6.2%; breakage 15%	Pilot/prototype	Chen et al., 2020
Carcass weight estimation for yield/portioning support	2D images + 3D statistical shape prior	136,472 images from a real production site; 3D shape model constructed from 45 carcass scans	Internal model evaluation on production-site images; independent plant validation not reported	Mean absolute percentage error 3.47%; algorithm reported as real-time capable	Commercial-context / offline	Jørgensen et al., 2019
Foreign-material detection on poultry meat	NIR hyperspectral imaging + semisupervised deep learning	∼879,000 clean-meat spectral responses for training; testing included 30 foreign-material types at 2 × 2 and 5 × 5 mm	Controlled laboratory test; no prospective commercial-line validation	Pixel precision 100%, recall >93%, F1 96.8%, balanced accuracy 96.9%; object-level accuracy 96.5%. Later compression study used simulated 140–250 birds/min scenarios	Laboratory	Campos et al., 2023; Khan et al., 2025
Wooden-breast detection and grading	Industrial NIR spectroscopy	Calibration n = 197 fillets; industrial test n = 79; online scanner then measured three flocks (9,063; 6,330; 10,483 fillets)	Independent industrial test followed by online commercial-line use; multi-plant validation not reported	99.5–100% calibration accuracy and 100% test accuracy; large-flock online incidence measurement demonstrated	Commercial-line	Wold et al., 2017
Further-processed product quality-deviation detection	Overhead RGB imaging + YOLOv12	Real ready-to-eat poultry line; 2,000 labeled images at each of 4 stages (forming, coating, frying, cooking), collected over multiple dates	Randomized image-level 80/20 split within production-line dataset; no independent plant test	Step-specific peak mAP50–95 ∼0.50–0.60; combined-stage model 0.7331 ± 0.0040; rare classes frequently misclassified	Commercial-context / offline	Einsiedel et al., 2026
Microbial-quality and spoilage assessment	Multispectral imaging and FTIR + regression/classification models	402 chicken thigh fillets under 8 isothermal and 2 dynamic storage profiles	Controlled laboratory modeling within experimental dataset; no plant-level external validation	MSI RMSE: 0.987 log CFU/cm² (TVC) and 1.215 log CFU/cm² (*Pseudomonas*); MSI-SVM fresh/spoiled accuracy 94.4%	Laboratory	Spyrelli et al., 2021a
Fecal-contamination screening	Multispectral fluorescence imaging + machine learning	404 broiler carcasses; laboratory observations followed by processing-facility validation on 114 swab samples	Laboratory development + limited in-plant validation	Detected visually inapparent fecal contamination; fecal-source discrimination did not exceed 55.40% under the reported threshold; some invisible sites were *Salmonella*-positive	Pilot / in-plant validation	Black et al., 2025

Reported performance values are not directly comparable among studies because sample populations, class definitions, reference methods, data partitioning, and validation settings differed. “Commercial-context / offline” indicates use of real production-line or plant data without prospective validation of an integrated decision/actuation system; “Commercial-line” indicates prospective operation on an active commercial processing line. Laboratory or internally validated performance should not be interpreted as evidence of commercial-line robustness.

**Abbreviations:** AP, average precision; F1, F1 score; FTIR, Fourier-transform infrared spectroscopy; MSI, multispectral imaging; NIR, near-infrared; RMSE, root-mean-square error; TVC, total viable count; VIS, visible.

***Live-bird receiving and welfare-linked monitoring*.** Information collected before slaughter could help processors anticipate carcass-weight variation, welfare concerns, condemnation risk, and the need for equipment adjustment. Computer vision has been used extensively on farms to monitor bird distribution, activity, posture, feeding, and abnormal behavior. These systems offer continuous and noncontact observation, but their accuracy is affected by crowding, overlapping birds, variable lighting, and the difficulty of obtaining consistent behavioral labels (Okinda et al., 2020).

Applying the same approach during unloading, shackling, or stunning is reasonable, but direct processing-plant evidence remains limited. Video systems could potentially identify unusual movement, improper bird positioning, or repeated handling problems. Nevertheless, most published poultry-welfare models were developed in controlled farm environments rather than in noisy, crowded, rapidly moving slaughter areas. Farm-level accuracy should therefore not be assumed to transfer directly to plant conditions (Jia et al., 2026; O’Bryan et al., 2026).

AI-based welfare monitoring is best considered a decision-support tool. It could help plant personnel identify patterns that are difficult to observe continuously, but it should not replace trained welfare personnel without independent validation against recognized welfare outcomes. Further studies should connect video-based predictions with measures such as stunning effectiveness, carcass damage, inspection findings, and processing conditions across multiple flocks and days.

**Slaughter, scalding, defeathering, and process-related defects**. Slaughter, scalding, and defeathering equipment generally operates within predefined settings, although incoming birds vary considerably. Insufficient scalding or picking may leave feathers, whereas excessive mechanical action may contribute to skin damage, exposed tissue, bruising, or broken parts. Cameras positioned after these operations could therefore provide more than product grading: repeated detection of a particular defect could serve as an early warning that a machine or process setting is drifting away from its desired condition.

Recent deep-learning systems demonstrate the potential of this approach. CarcassFormer uses a transformer-based architecture to detect, segment, and classify poultry-carcass defects simultaneously. Its dataset included 7,321 images containing single and multiple carcasses and represented defects associated with production, transportation, stunning, scalding, picking, and other equipment-related conditions. Unlike whole-image classification, the system identifies where a defect occurs, making the output more useful for trimming, sorting, and process diagnosis (Tran et al., 2024).

Lai & Kuo (2023) similarly developed a two-stage system for native chickens in which YOLOv7 first located the wings, breast, back, and legs, after which a classification model evaluated the individual regions for defects. The reported mean average precision for body-part detection was 98.9%, and defect-classification accuracy was 94.2%. This body-region approach is valuable because variation in skin color, carcass size, and breed can make a single whole-carcass model unreliable.

These results are promising, but they do not yet establish routine industrial performance. CarcassFormer was evaluated primarily as an annotated-image benchmark, and strong detection or segmentation scores do not show that the system can maintain accuracy over a complete production shift. Images collected close together may also share lighting, background, and flock characteristics, making the test set less independent than it appears. Commercial validation should therefore include different flocks, shifts, plants, equipment settings, carcass sizes, and seasons. It should also report missed-defect rates, false rejections, processing speed, and the economic consequence of each error.

The most useful application may be trend-based process monitoring. One missed feather on a single carcass may be unimportant, but a rapid increase in retained feathers across many carcasses could indicate a scalding or picker problem. This converts AI from a simple pass–fail classifier into a process-control aid. Nevertheless, evidence is still needed that responding to these alerts actually reduces defects, product loss, or equipment downtime.

***Evisceration and carcass inspection***. AI can support evisceration by locating anatomical structures and adapting equipment to individual carcasses. Using color-space transformations and active-contour segmentation, Chen & Wang (2018) obtained visceral-contour recognition rates of 93.3% for Three-Yellow chickens and 86.7% for Cherry Valley ducks. The difference between poultry types indicates that anatomical-recognition models may not transfer reliably across breeds or species. Subsequent machine-vision systems improved viscera segmentation and positioning, including reported recognition rates approaching 99% under experimental conditions (Chen et al., 2021). Three-dimensional point-cloud data combined with a genetic-algorithm-optimized wavelet neural network have also been used to predict internal visceral dimensions from external carcass measurements (Zhu et al., 2025).

Nevertheless, accurate localization does not guarantee successful evisceration. In a robotic evaluation, Chen et al. (2020) reported carcass and incision localization errors of only a few pixels, but visceral residue and breakage rates remained 6.2% and 15%, respectively. This discrepancy demonstrates that image-level accuracy may overstate operational performance because small positioning errors can cause economically or hygienically important damage. Evaluations should therefore report organ damage, residual material, contamination, cycle time, and recovery from failed positioning, not segmentation accuracy alone.

Automated carcass inspection has stronger commercial evidence. A line-scan spectral system inspected more than 100,000 carcasses at 140 birds/min and correctly classified over 99% of wholesome and over 96% of unwholesome carcasses (Chao et al., 2011). Hyperspectral and multispectral systems have also detected fecal and ingesta contamination at processing-line speeds (Park et al., 2002; Yoon et al., 2011). More recently, multispectral fluorescence imaging and machine learning detected contamination that was not visually apparent (Black et al., 2025), while CarcassFormer simultaneously localized, segmented, and classified processing defects using 7,321 images (Tran et al., 2024). However, fluorescence indicates contamination risk rather than pathogen presence, and strong image metrics do not establish performance across plants, shifts, carcass orientations, or rare conditions. AI is therefore more defensible as a continuous presorting and decision-support system, with uncertain or high-risk carcasses referred to trained inspectors.

***Chilling and antimicrobial process control*.** Chilling involves nonlinear relationships among carcass weight, initial temperature, water temperature, agitation, flow rate, residence time, and equipment configuration. Artificial neural networks have consequently been used to predict carcass exit temperature from industrial process data. Klassen et al. (2009) modeled immersion chilling using carcass and chiller variables, while Silveira et al. (2014) obtained a regression coefficient of 0.9265 for industrial prediction of final carcass temperature. Neural networks have also predicted water uptake during immersion chilling using variables related to heat transfer, mass transfer, and initial carcass characteristics (Martins et al., 2011). These models demonstrate useful predictive capability, but they were not evaluated as autonomous controllers that adjusted operating conditions prospectively.

Antimicrobial control is similarly affected by interacting process conditions. Kroft et al. (2024) found that peroxyacetic acid (PAA) efficacy against *Salmonella* on chicken wings depended primarily on PAA concentration and solution temperature, whereas pH and treatment time were not significant under the evaluated conditions. A meta-analysis further demonstrated considerable variation in microbial reduction among chilling and post-chilling interventions, emphasizing that antimicrobial performance cannot be inferred from concentration alone (Leone et al., 2024). Ciluveru et al. (2026) combined experiments with mathematical modeling and found that total dissolved solids predicted PAA decay more consistently than chemical oxygen demand. Their model also showed that organic loading altered the balance between bacterial shedding and PAA-mediated inactivation.

Overall, current studies provide predictive models rather than validated closed-loop control. Before deployment, models must be tested prospectively across carcass sizes, organic loads, plants, seasons, and chiller configurations. A safe control system should combine continuous measurements of temperature, flow, antimicrobial concentration, and water quality with constrained operating limits. Performance should be evaluated through final carcass temperature, antimicrobial residuals, microbial reduction, water absorption, energy and water use, and frequency of unsafe or out-of-specification predictions.

***Cut-up, deboning, and yield optimization.*** Cut-up and deboning are difficult to automate because carcasses are deformable and anatomically variable, while many cutting boundaries are not externally visible. Hu et al. (2012) proposed an adaptive deboning framework that combined image-based carcass characterization, generation of a nominal cutting trajectory, and force feedback to correct deviations during shoulder cutting. Vision-guided water-jet systems have similarly used fillet shape and thickness to optimize portioning patterns for predetermined weights and dimensions (Heck, 2006). However, commercial system descriptions provide limited independent evidence regarding yield, bone fragments, sanitation, and long-term reliability.

Computer vision can also estimate carcass and cut weights before physical separation. Jørgensen et al. (2019) evaluated 136,472 production images and obtained a mean absolute percentage error of 3.47% using three-dimensional prior information. However, the underlying shape model was constructed from only 45 three-dimensional carcass scans, illustrating that a large image count does not necessarily represent broad biological diversity. Nyalala et al. (2021) reported coefficients of determination ranging from 0.913 for drumstick weight to 0.998 for whole-carcass weight. These results are promising, but (R^2^) does not indicate how frequently products would be assigned to the wrong commercial weight category.

Vision can additionally quantify residual meat and cutting defects. Usher & Daley (2013) developed a system that measured residual meat on deboned frames in less than one second and achieved up to approximately 90% correlation with manual yield measurements. A newer vision-guided cutting system reported 98.86% detection mAP and 22.2-ms image inference, yet residual rates after robotic cutting remained approximately 11.7%–11.8% (Mei et al., 2025). Likewise, laser-based three-dimensional portioning produced chicken-breast weight errors of 4.53%–5.96%, but the identified cutting lines were implemented manually rather than through a fully integrated commercial cutting system (Thongprasith et al., 2025). These findings reinforce that perception accuracy is not equivalent to cutting performance. Industrial validation should prioritize recovered yield, weight-category accuracy, bone-fragment incidence, product damage, throughput, sanitation compatibility, downtime, and performance across independent flocks and plants.

***Detection and classification of breast-muscle abnormalities*.** Breast-muscle abnormalities are promising but technically demanding targets for AI because their detectable characteristics differ considerably. WS is primarily identified from surface striations, whereas WB alters fillet shape, stiffness, composition, and optical scattering. SM is characterized by reduced structural integrity, while deep pectoral myopathy (DPM) may remain concealed within the *pectoralis minor* (Tushar et al., 2026). Hemorrhage and bruising produce color and spectral changes that can overlap with normal processing variation. Therefore, a single RGB model is unlikely to classify all abnormalities reliably, particularly when multiple conditions coexist.

Near-infrared spectroscopy provides some of the strongest industrial evidence for WB detection. Wold et al. (2017) trained an NIR model with 197 fillets and obtained 100% classification in an industrial test set of 79 fillets. The online scanner subsequently evaluated three commercial flocks containing 6,330–10,483 fillets each. This large-scale operation is an important strength, although the independent test set was comparatively small and validation was not reported across multiple plants. Geronimo et al. (2019) obtained 91.8% accuracy using computer vision and 97.5% using NIR spectroscopy, but only 80 fillets were evaluated. Similarly, portable NIR models classified normal, WS, and WB-associated samples with 92–93% accuracy, but WB and combined WB/WS fillets could not be separated and were consequently merged into one class (de Carvalho et al., 2020). Thus, high broad-class accuracy does not necessarily indicate reliable discrimination of overlapping abnormalities.

Imaging can detect WB before or after deboning by measuring carcass or fillet conformation. A model based on commercial broiler-carcass dimensions achieved approximately 84% validated accuracy across birds differing in sex, strain, and age (Caldas-Cueva et al., 2021). A high-speed side-view system subsequently measured fillet deformation and exceeded 95% binary WB-detection accuracy (Yoon et al., 2022). Lu et al. (2024) achieved 100% classification accuracy for severe WB samples using a partial least squares prediction model applied to bioelectrical impedance (BIA) characteristics. Whereas Siddique et al. (2022) found 88.89% accuracy for severe WB. These methods are operationally attractive because they use relatively simple geometric information. However, carcass dimensions and fillet bending are indirect indicators that may also vary with body weight and product orientation. Their robustness therefore depends on whether these sources of variation were represented during validation.

More advanced imaging can reveal subtle surface and subsurface differences. Structured-illumination reflectance imaging combined with deep features achieved 96.6% accuracy when separating normal from WS-affected fillets, but accuracy declined to 83.4% when three severity categories were considered (Olaniyi et al., 2023). Light-scattering imaging similarly achieved 92.3% accuracy for binary WB detection, with selected handcrafted features outperforming deep-learning features (Cai and Lu, 2025). This finding is important because it demonstrates that deep learning is not automatically superior when physically meaningful optical features are available.

Evidence for SM detection is less mature. Using 300 fillets from three flocks, BIA combined with support-vector machines produced test accuracies of 71.0%, 60.0%, and 81.5% for normal, moderate, and severe WB, respectively. A neural network separated normal fillets with and without SM with 100% training accuracy, but independent SM classification was substantially weaker, indicating possible overfitting and inadequate class separation (Siddique et al., 2021). Muñoz-Lapeira et al. (2025) achieved 94.0% balanced accuracy in discriminating SM from other myopathies using VIS-NIR hyperspectral imaging (386–1016 nm) by exploiting spectral differences related to myoglobin content and water binding.

AI has also been applied to blood-related defects. Hyperspectral–CNN models achieved mean average precision values of 0.916–0.990, with inference times of 41.8–58.2 ms (Duan et al., 2024). Nevertheless, fast inference is not equivalent to commercial throughput because image acquisition, carcass tracking, communication, and rejection must also occur within the available processing time. Comparable poultry-specific validation remains scarce for DPM and ultrasound-based abnormality detection; these should therefore be presented as research priorities rather than established industrial applications.

**Fig. 4:**
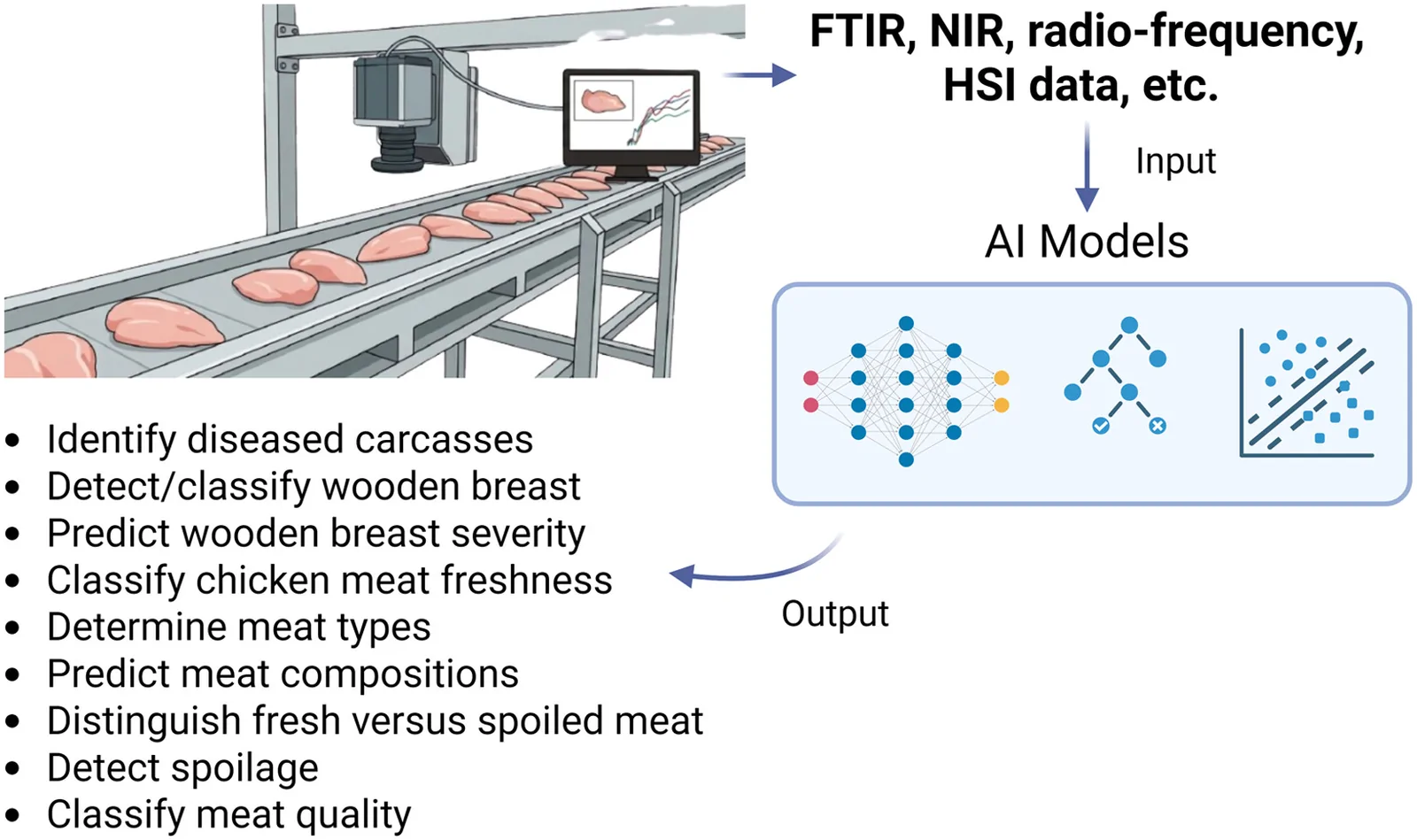
General framework for AI-assisted nondestructive assessment of poultry meat and carcasses. Adapted from Jia et al. (2026), created with BioRender.

***Nondestructive prediction of meat-quality traits*.** Nondestructive quality prediction differs fundamentally from defect classification. Classification assigns a product to a category, whereas regression attempts to estimate a continuous laboratory value such as pH, drip loss, shear force, or protein content. Consequently, a high correlation is insufficient evidence of practical usefulness. Models should be evaluated using independent prediction error, bias, prediction intervals, and their ability to make the intended commercial decision. The general framework for AI-assisted nondestructive assessment of poultry carcasses and meat is presented in Fig. 4.

Color and pH are among the most frequently modeled traits because visible spectra are directly affected by meat pigments and light scattering. Using VNIR hyperspectral imaging, Jia et al. (2017) obtained an independent-test (R_p_^2^) of 0.73 and root mean square error for independent test set (RMSEp) of 0.29 pH units, despite substantially stronger performance during model validation. This difference illustrates why cross-validation results should not be interpreted as equivalent to performance on genuinely new samples. Furthermore, if only surface color is required, conventional RGB imaging or an inline colorimeter may be more economical than hyperspectral imaging.

Water-holding capacity is more difficult because it reflects pH, protein functionality, muscle structure, and postmortem conditions rather than a single optical property. Fusion of spectral and image-texture information produced (Rp=0.80) and RMSEp=0.80 for drip loss, but performance was weaker for expressible fluid (Rp=0.56, RMSEp=2.10) and salt-induced water gain (Rp=0.69, RMSEp=18.04) (Yang et al., 2018). Thus, one sensor may predict one water-holding measurement reasonably while performing poorly for another, and these measurements should not be treated as interchangeable.

Texture prediction presents similar challenges. Hyperspectral models for raw WB fillets predicted MORS peak count with (Rp=0.915) and RMSEp=2.26, but three-class WB-severity accuracy was only 77.8% (Liu et al., 2025). Spectral associations with texture may arise from water, protein, collagen, or myopathy-related structural changes; therefore, models calibrated on one population may fail when these relationships change. Prediction against an instrumental shear test also demonstrates agreement with that test, not necessarily tenderness perceived by consumers.

NIR and hyperspectral techniques have also been applied to composition. Early NIR models produced strong calibration relationships for moisture, protein, and fat in chicken muscles, but the experiment included only 48 birds and obtained its strongest results from defrosted, minced samples (Cozzolino et al., 1996). Performance on homogenized meat cannot be assumed for intact fillets because surface moisture, fiber orientation, and compositional heterogeneity affect spectral measurements. Hyperspectral imaging predicted hydroxyproline, a collagen-related marker, with (Rp=0.874) and RMSEp=0.046, demonstrating feasibility but not yet plant-level robustness (Xiong et al., 2015a).

Oxidation and freshness can be modeled because storage produces progressive chemical and microbiological changes (Mostafa et al., 2025). A hyperspectral model predicted TBARS with (Rp=0.944) and RMSEP=0.081 (Xiong et al., 2015b). Similarly, NIR prediction of dominant spoilage organisms achieved (Rp=0.954) and RMSEp of approximately 0.40 log CFU/g (Jiang et al., 2021). However, models may partly learn storage time or temperature rather than oxidation or microbial load itself. Independent-batch validation provides a more demanding assessment: FT-IR models predicted chicken-surface microbial counts with an error of approximately 0.85 log CFU/cm², whereas an MSI model produced an independent-batch error of 1.25 log CFU/cm² (Spyrelli et al., 2021a). An error near one logarithmic unit represents roughly a tenfold difference in microbial concentration and may be acceptable for screening but not precise microbiological verification.

***Further-processed poultry products***. Further processing introduces additional sources of variation because product quality depends simultaneously on raw-material composition, formulation, forming, coating, thermal processing, and packaging. AI can integrate images, product dimensions, temperatures, equipment settings, and formulation records to identify deviations earlier than end-product sampling. However, academic evidence remains less extensive than for carcass inspection or breast-myopathy detection, and many reported industrial applications remain proprietary (Jia et al., 2026; Zatsu et al., 2024).

The strongest recent evidence comes from an actual ready-to-eat poultry-processing line. Einsiedel et al. (2026) collected 2,000 annotated images at each of four stages—forming, coating, frying, and cooking and trained step-specific YOLOv12 models to detect quality deviations. The individual models achieved peak mAP (50–95) values of approximately 0.50–0.60, whereas a combined model covering all stages reached (0.7331 ± 0.0040). Nevertheless, performance was strongly dependent on class frequency, with uncommon defects frequently misclassified. Thus, combining production stages may improve overall learning, but it does not remove the need for targeted collection of rare yet economically or safety-relevant defects (Einsiedel et al., 2026).

Computer vision can quantify product shape, dimensions, color, coating coverage, and surface irregularities after forming or portioning. Yuangyai et al. (2013) developed an image-based system for chicken nuggets that measured 11 characteristics related to meat color, coating color, porosity, adhesion, and surface area. Principal-component analysis reduced these measurements to four dimensions, supporting more objective identification of abnormal products. However, the study primarily established a measurement framework; it did not demonstrate autonomous rejection, equipment adjustment, or reliable performance across multiple production lines (Yuangyai et al., 2013).

Image information can also serve as an indirect predictor of texture. Qiao et al. (2007) extracted five image-texture indices from chicken nuggets collected at different frying stages and used a multilayer feed-forward neural network to predict maximum load, energy to break point, and toughness. Correlations between predicted and mechanically measured values exceeded 0.84. Similarly, ultrasonic velocity showed a strong relationship with sensory crispness in breaded fried nuggets, suggesting that acoustic information could complement surface imaging (Antonova et al., 2003; Qiao et al., 2007). Nevertheless, correlations with instrumental texture or trained-panel scores do not establish prediction of consumer liking, and both relationships may change with coating formulation, frying oil, product temperature, and post-fry holding.

Coating and breading are particularly difficult because surface coverage does not fully describe coating adhesion, internal porosity, moisture migration, or oil uptake. X-ray micro-computed tomography has demonstrated that frying conditions alter the three-dimensional pore structure of breaded chicken nuggets, but such detailed imaging is not presently suitable for routine high-speed inspection (Adedeji and Ngadi, 2011). A practical AI system may therefore combine rapid RGB or spectral imaging with occasional destructive measurements of coating pickup, adhesion, moisture, fat, and crispness. Models trained only on visual appearance could otherwise accept products that look uniform but have inadequate coating functionality.

Cooking is a higher-consequence application because surface browning does not confirm that a safe internal temperature has been reached. Ibarra et al. (2000) combined infrared images with a multilayer neural network to estimate the internal temperature of cooked chicken. The model produced a standard error of approximately ±1.01°C over 0–540 s after cooking and ±1.07°C during the first three minutes. Ma & Tao (2005) subsequently combined infrared surface temperatures, three-dimensional geometry, and time-lagged images; their neural-network model achieved a mean absolute error of 1.54°C and a mean absolute percentage error of 2%. These results demonstrate the value of sensor fusion because product thickness strongly affects heat transfer. However, the studies were conducted under controlled or pilot-scale conditions, and temperature prediction must not be equated automatically with microbial lethality. Commercial validation should include different formulations, product sizes, belt loading patterns, ovens, and overlapping or folded products.

AI-supported inspection can also identify malformed products, exposed meat, excessive or insufficient browning, coating loss, foreign material, package damage, incorrect labels, and unreadable date codes. Yet RGB systems are primarily sensitive to visible surface deviations; internal bone fragments, embedded contaminants, seal-channel contamination, and incorrect formulation may require X-ray, hyperspectral, thermal, or production-record data. Poultry-specific peer-reviewed evidence for AI-based package and label verification remains particularly limited, despite broader food-industry interest in computer vision and optical-character-recognition systems (Jia et al., 2026; Zatsu et al., 2024).

***Artificial Intelligence in Poultry Meat Packaging***. Packaging is a critical control point in poultry processing because package integrity, headspace composition, storage temperature, labeling accuracy, and product freshness collectively determine safety, shelf life, and consumer acceptance. Conventional packaging inspection generally relies on fixed machine-vision rules, periodic destructive testing, and human observation. These approaches may identify obvious defects but are less effective when seal contamination, film deformation, condensation, variable product orientation, or subtle spoilage indicators create complex and nonlinear patterns. AI, particularly computer vision, machine learning, deep learning, and sensor fusion, can provide more adaptive inspection and decision support by integrating visual, spectral, chemical, and cold-chain information.

One of the most immediate applications is automated inspection of trays, films, seals, labels, and product presentation. CNN and related vision models can identify incomplete seals, wrinkles, punctures, contamination within the sealing region, damaged trays, incorrect fill levels, misplaced absorbent pads, and poorly positioned poultry portions. Multispectral or hyperspectral imaging may be especially useful where transparent films, reflective surfaces, or food residues make conventional RGB inspection unreliable. Benouis et al. (2021), for example, combined hyperspectral image fusion with deep-learning models to classify normal and faulty food-tray seals, with the CNN achieving approximately 90% accuracy. Although the study was not poultry-specific, the approach is directly relevant to poultry trays because meat or purge trapped within the sealing interface can compromise package integrity while remaining difficult to detect under visible illumination. However, an accuracy near 90% may still be inadequate for high-throughput commercial use when rare seal failures have serious safety and economic consequences.

AI can also strengthen label and traceability control. Computer-vision and optical-character-recognition systems can verify product identity, net weight, use-by dates, barcodes, lot codes, allergen declarations, cooking instructions, and alignment between the packaged product and its label. These systems could integrate information from production schedules, formulation records, weighing equipment, and enterprise databases to detect mismatches before cases leave the packaging line. Such integration is particularly important for further-processed poultry products containing marinades, breading systems, or multiple allergens. Nevertheless, the literature specifically validating AI-based label verification in poultry plants remains limited. Most available studies address general manufacturing or food packaging, and their performance may not transfer directly to wet, reflective, rapidly moving poultry packages.

A more advanced application is the integration of AI with intelligent packaging for freshness and shelf-life assessment. Sensors or indicators incorporated into packages may respond to temperature history, pH changes, oxygen, carbon dioxide, ammonia, hydrogen sulfide, volatile organic compounds, or microbial metabolites (Mostafa et al., 2025). ML models can interpret these multivariate signals and estimate freshness condition or remaining shelf life more dynamically than a fixed expiration date (Li et al., 2023). This is particularly relevant to poultry meat because spoilage kinetics are influenced by initial microbial load, packaging atmosphere, temperature fluctuations, product form, and processing history.

Evidence from chicken-freshness studies illustrates this potential. Taheri-Garavand et al. (2019) combined computer-vision features with a genetic algorithm and artificial neural network to predict chicken-meat freshness during refrigerated storage, reporting a high correlation between predicted and reference freshness values. Xiong et al. (2023) applied transfer learning to electronic-nose data collected from chicken breasts stored at 4°C and reported 99.70% classification accuracy using a ResNet model, exceeding the performance of several conventional machine-learning methods. Similarly, Weng et al. (2020) fused electronic-nose, computer-vision, and artificial-tactile measurements for pork and chicken and obtained stronger freshness prediction than with individual sensing systems; the chicken model achieved an R² of 0.94 and a root-mean-square prediction error of 0.98 for total volatile basic nitrogen. These findings suggest that multimodal AI can capture complementary spoilage signals that may be missed by a single indicator.

Reported accuracies for AI-based freshness prediction should be interpreted cautiously because many studies use samples from a single source, controlled storage conditions, and random data splitting. Under these conditions, models may partly learn storage-time progression or batch-specific patterns rather than the wider biological and operational variability encountered commercially. Freshness categories based on storage duration, sensory scores, or chemical indicators also do not necessarily represent pathogen risk or consumer acceptability. External validation across products, plants, packaging systems, seasons, initial microbial loads, and temperature-abuse conditions is therefore necessary.

AI could support dynamic packaging by integrating headspace gases, temperature history, film properties, package images, and shelf-life data to detect leakage, estimate remaining shelf life, optimize inventory, and improve traceability. Connected packaging systems using radio-frequency identification, wireless sensors, time–temperature indicators, and Internet-of-things platforms may enable real-time monitoring across the cold chain (Madhu, 2025; Sagar and Rani, 2026; Yu et al., 2026). However, sensor cost, drift, matrix-specific responses, regulatory requirements, and additional packaging waste remain important barriers.

### AI for poultry food safety

**Fig. 5:**
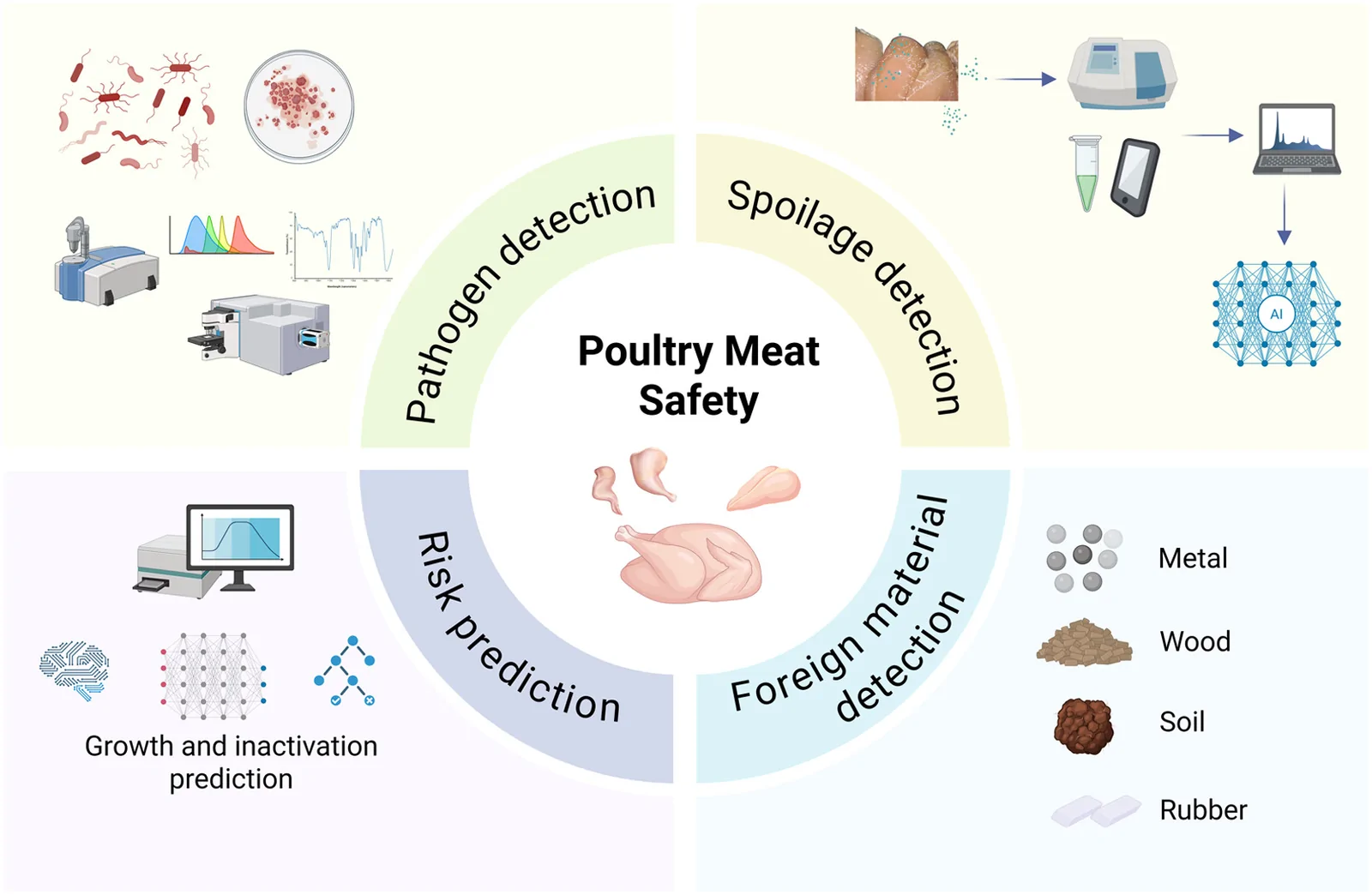
Major applications of artificial intelligence in poultry-meat safety assessment. Created with BioRender.

AI-assisted poultry food-safety assessment encompasses microbial contamination detection, sanitation monitoring, spoilage and microbial-load prediction, sensor-based screening, and predictive risk modeling, as summarized in Fig. 5.

***Microbial contamination detection*.** Some of the strongest pathogen-specific evidence involves automated identification after microbial culture. Hyperspectral imaging differentiated *Campylobacter* colonies from 16 other poultry-associated organisms with more than 99% reported accuracy (Yoon et al., 2009). Similarly, models developed for *Salmonella Enteritidis* and *S.* Typhimurium achieved approximately 98–99% accuracy when classifying colonies from spiked poultry-carcass rinses cultured on selective agar (Seo et al., 2014). These systems could accelerate presumptive colony screening and reduce analyst subjectivity. However, they still require sampling, plating, and incubation; consequently, they automate laboratory interpretation rather than detect pathogens directly on moving carcasses.

Imaging can also identify contamination associated with pathogen risk. Multispectral fluorescence imaging detected visible and visually undetected fecal material on 404 broiler carcasses, and some visually undetected locations were positive for *Salmonella*. Nevertheless, the model could not reliably identify the gastrointestinal source of the contamination, and plant validation was limited to 114 swabs (Black et al., 2025). Earlier visible–NIR spectroscopy distinguished fecal or ingesta residues from poultry-equipment surfaces with 100% sensitivity to the applied contaminants, but specificity for clean equipment was approximately 92.5–95% (Chao et al., 2008). Such systems detect organic contamination requiring corrective action, but fecal or residue detection remains a risk proxy rather than pathogen confirmation.

AI has been evaluated more extensively for spoilage organisms and total microbial load. Using 402 chicken-thigh fillets, multispectral imaging predicted total viable counts and *Pseudomonas* counts with RMSE values of 0.99 and 1.22 log CFU/cm², respectively, while an SVM classified fresh and spoiled samples with 94.4% accuracy (Spyrelli et al., 2021a). FTIR spectra combined with feature selection and SVM regression predicted microbial groups in chicken liver with RMSE values of 0.66–0.95 log CFU/g (Dourou et al., 2021).

Very high freshness-classification accuracies also require careful interpretation. An electronic nose and transfer-learning model achieved 99.7% accuracy for chicken breasts stored at 4°C for 1–7 days (Xiong et al., 2023), while NIR models classified initiation, propagation, and spoiled stages with accuracies of 93.7–98% (Black et al., 2025). However, models developed from controlled storage sequences may learn systematic changes associated with storage day, temperature, or batch rather than microbial contamination alone. External validation should therefore incorporate different initial microbiota, packaging systems, temperature fluctuations, processing plants, and product formulations.

Environmental monitoring offers a complementary strategy. Pathogen loads in farm samples have been associated with loads in carcass rinses from the same commercial flocks (Berghaus et al., 2013), and random-forest models using management and processing variables predicted *Salmonella* occurrence in pastured-poultry samples with AUC values exceeding 0.83 (Hwang et al., 2020). Commercial bio-mapping across three broiler plants further demonstrated that contamination patterns and intervention effectiveness differed among facilities (Chavez-Velado et al., 2024). These findings support plant-specific early-warning models, but historical associations cannot replace direct microbiological verification.

***Technologies used*.** Hyperspectral and multispectral imaging combine spectral composition with spatial localization. They can identify contaminated regions, characterize colonies, and potentially inspect large product areas without physical contact (Yoon et al., 2009; Black et al., 2025). Hyperspectral instruments are particularly valuable for discovering informative wavelengths, whereas reduced-band multispectral systems are more compatible with commercial speed and cost. Their principal weaknesses include large data volumes, sensitivity to lighting and surface moisture, and limited pathogen specificity when biochemical signals overlap.

FTIR and NIR spectroscopy are simpler for rapid microbial-quality estimation but generally provide less spatial information. Their models primarily detect biochemical changes caused by microbial metabolism rather than microbial cells themselves (Dourou et al., 2021; Spyrelli et al., 2021a). Consequently, spectral relationships can change with product composition, marinades, myopathies, packaging, temperature, or dominant spoilage organisms. Reduced-wavelength systems should therefore be developed only after external validation confirms that selected bands remain informative across batches and plants (Siddique et al., 2024).

Fluorescence imaging is especially promising for sanitation verification because organic residues and bacterial biofilms can fluoresce under ultraviolet excitation. Fluorescence hyperspectral imaging classified *E. coli* and *Salmonella* biofilms on stainless-steel and polyethylene coupons with sensitivity and specificity generally exceeding 90% (Lee et al., 2021). Poultry-specific research also showed that color and fluorescence measurements combined with ML could distinguish clean surfaces from surfaces carrying diluted chicken residues (Griscom et al., 2025).

Biosensors provide greater biological specificity by using antibodies, aptamers, molecularly imprinted polymers, or other recognition elements. A quantum-dot immunoassay detected *Salmonella* and *Campylobacter* in poultry samples at approximately 30–50 CFU/mL within two hours (Wang et al., 2014). A surface-imprinted sensor detected *C. coli* and *C. jejuni* in poultry-associated samples within one hour, although detection limits differed substantially between species (Givanoudi et al., 2021). An impedance-based microfluidic device also reported rapid *Salmonella* detection in raw chicken rinsate (Almalaysha et al., 2024). However, these studies mainly demonstrate sensor performance rather than mature AI integration. A biosensor should not be described as AI-enabled merely because its output is digital; AI must provide a validated improvement in signal interpretation, multiplexing, error detection, or decision-making.

Electronic noses detect volatile mixtures generated during spoilage and can be combined with neural networks or transfer learning (Xiong et al., 2023). They are rapid and potentially inexpensive, but sensor drift, humidity, packaging atmosphere, marinades, and cleaning chemicals can alter volatile profiles. Moreover, volatile-based freshness prediction cannot establish pathogen absence because pathogens may be present without producing a detectable spoilage odor.

Microbiological, environmental, and process records provide another important data source. AI can combine flock history, carcass-rinse results, sanitation records, antimicrobial conditions, equipment location, and production time to identify elevated-risk periods or contamination routes (Berghaus et al., 2013; Chavez-Velado et al., 2024). Sensor fusion may further combine spectral, imaging, volatile, temperature, and microbial data; however, multisensor studies show that additional sensors do not always improve prediction consistently (Spyrelli et al., 2022). Each added sensor increases calibration, sanitation, maintenance, and data-integration requirements.

***Predictive microbial risk modeling and AI-enhanced HACCP*.** Predictive microbial modeling links monitoring data with preventive food-safety decisions by integrating time–temperature history, product characteristics, antimicrobial exposure, processing conditions, and microbiological records to estimate microbial growth, inactivation, cross-contamination, and shelf life. However, mechanistic models and quantitative microbial risk assessment (QMRA) remain important benchmarks, and greater algorithmic complexity is justified only when ML approaches improve external prediction, uncertainty estimation, or operational decision-making.

Poultry-specific studies demonstrate the potential of these approaches but also their limited validation domains. Oscar (2016) developed a neural-network model for eight *Salmonella* serotypes in ground chicken, achieving acceptable prediction-zone proportions of 0.948 for training data and 0.988 for independent testing. In chicken liver, FTIR spectroscopy combined with feature selection and support-vector regression predicted microbial populations with errors of 0.664–0.949 log CFU/g (Dourou et al., 2021). Multispectral imaging similarly predicted total viable and *Pseudomonas* counts on chicken thighs, while an SVM classified fresh and spoiled samples with 94.4% external-validation accuracy (Spyrelli et al., 2021a). Xu et al. (2022) also integrated temperature, humidity, gas, physicochemical, and sensory data through IoT sensors and a neural network, obtaining quality-classification accuracy above 90.5%. Nevertheless, these systems primarily support freshness and shelf-life assessment; prediction errors near one logarithmic unit remain microbiologically relevant, and spoilage indicators do not establish the absence of specific pathogens.

At the processing-chain level, QMRA models can estimate changes in contamination across successive operations. Nauta et al. (2005) modeled *Campylobacter* removal, inactivation, and cross-contamination, Pradhan et al. (2007) predicted *Salmonella* prevalence and concentration from live-bird arrival through chilling, and Dogan et al. (2019) evaluated alternative *Campylobacter* interventions. Such models can identify influential processing stages and compare control strategies, but their conclusions depend on assumptions concerning initial prevalence, transfer coefficients, intervention efficacy, and dose–response relationships. Substantial heterogeneity among intervention studies further limits the transferability of estimated microbial reductions across plants and processing conditions (Leone et al., 2024).

AI-enhanced HACCP could integrate continuous measurements of temperature, antimicrobial concentration, exposure time, pH, flow rate, line speed, and equipment status to identify developing deviations, alert operators, trace affected product, and document corrective responses. However, AI does not replace established HACCP requirements. Critical limits, corrective actions, verification procedures, and documentation must remain scientifically validated and consistent with Codex and FSIS requirements (WHO, 2023; USDA-FISS, 2025). Current AI applications should therefore be considered decision-support tools rather than autonomous systems capable of redefining critical limits or product disposition.

The principal limitation is that many models infer microbial risk indirectly from spectral, volatile, environmental, or process signals. These indicators may correlate with microbial deterioration without directly measuring *Salmonella* or *Campylobacter*, and low pathogen prevalence can produce misleadingly high overall accuracy despite unacceptable false-negative rates. Evaluation should therefore emphasize pathogen-specific sensitivity, specificity, predictive values, calibration, detection limits, and the proportion of unsafe samples classified as safe. Validation should also account for uncertainty in destructive microbiological reference methods, naturally contaminated products, strain diversity, low concentrations, mixed microbial populations, and realistic production and cold-chain variation. Model structure and dose–response assumptions must be examined carefully because they can materially influence risk estimates (Zagmutt and Pouillot, 2022).

### Challenges, Limitations, and Barriers to the Responsible Implementation of Artificial Intelligence in Poultry Processing

AI has demonstrated considerable potential for poultry-carcass inspection, foreign-material detection, meat-quality prediction, and defect classification. However, current research establishes technical feasibility more convincingly than industrial reliability. High performance reported for CarcassFormer, hyperspectral foreign-material detection, and blood-defect classification has largely been obtained using restricted datasets and controlled acquisition conditions that may not represent commercial variation among plants, seasons, equipment, products, and processing environments (Campos et al., 2023; Tran et al., 2024; Duan et al., 2024).

Many poultry-processing AI studies use samples from a single flock, facility, processing day, genetic line, sensor, or laboratory environment. Furthermore, image patches, spectral pixels, video frames, or augmented observations derived from the same carcass may be treated as independent samples, thereby inflating the apparent sample size and underestimating uncertainty (Campos et al., 2023; Kapoor and Narayanan, 2023; Jia et al., 2024). Class imbalance, limited representation of uncommon but consequential defects, inconsistent class definitions, and the absence of standardized poultry-processing benchmarks further restrict model comparison and generalizability (Liakos et al., 2025; Shi et al., 2021).

Prediction quality also depends on the reliability of the reference standard. Visual scoring may be influenced by assessor training, fatigue, disagreement, and subjective thresholds, whereas destructive measurements may not correspond spatially with the area assessed by imaging or spectroscopy. Laboratory assays also contain sampling and analytical uncertainty. Studies should therefore report annotation procedures, assessor agreement, sampling locations, replicate measurements, and assay precision (Shi et al., 2021; Jia et al., 2024; Tran et al., 2024).

Data leakage can occur when observations from the same carcass, flock, or production batch are distributed across training and testing datasets. Leakage may also arise when normalization, feature selection, or wavelength selection is conducted before data partitioning. Such practices can produce overly optimistic estimates of performance because the test data are not genuinely independent (Kapoor and Narayanan, 2023).

Validation should therefore occur at biologically and operationally meaningful levels, including the carcass, flock, processing day, season, camera, or plant. Model development should use nested validation, while a locked test set should remain untouched until development is complete. Independent and prospective commercial-line testing provides stronger evidence than random retrospective splitting of observations from the original dataset (Riley et al., 2024).

Performance reporting should extend beyond overall accuracy. Classification studies should provide class-specific sensitivity, specificity, precision, recall, balanced accuracy, confusion matrices, confidence intervals, and calibration. Regression studies should report prediction error, bias, residual patterns, and prediction intervals. Inference speed, hardware requirements, throughput, false-rejection rates, and procedures for handling uncertain or out-of-distribution samples are also essential for assessing industrial usefulness (Hüllermeier and Waegeman, 2021).

Commercial deployment is challenged by domain shifts caused by changes in lighting, moisture, carcass orientation, line speed, equipment configuration, sensor drift, genetic background, product type, and defect prevalence. Models should therefore be stress-tested under varied acquisition and production conditions, externally validated, and monitored after deployment using defined recalibration, updating, and rollback procedures (Jia et al., 2022; Shi et al., 2021).

Explainability tools may help determine whether models rely on biologically meaningful features or irrelevant artifacts, such as conveyor backgrounds, labels, shadows, or carcass positioning. However, predictive associations and post hoc visualizations should not be interpreted as evidence of causation. Explainability should be supported by feature ablation, background replacement, occlusion testing, error analysis, and expert evaluation (Rudin, 2019; Ghassemi et al., 2021).

Reproducibility remains limited by proprietary datasets, unavailable code, restricted algorithms, and incomplete reporting. Studies should provide preprocessing procedures, splitting identifiers, annotation protocols, software versions, random seeds, architectures, hyperparameters, and excluded observations. Independent replication remains necessary before broad claims of general applicability can be justified (Kapoor and Narayanan, 2023).

Industrial deployment requires more than an accurate model. Systems must include hygienically designed and rugged sensors, wash-down resistance, stable illumination, low-latency computing, cybersecurity, interoperability, automated calibration checks, and practical maintenance procedures. Integration with existing processing equipment and plant databases may be as challenging as model development itself (Jia et al., 2022; Liakos et al., 2025).

Economic assessments should compare installation, maintenance, calibration, software, storage, downtime, and prediction-error costs with measurable benefits such as labor savings, yield improvement, reduced rework, greater grading consistency, earlier equipment-failure detection, and improved food-safety control. Model accuracy alone provides insufficient justification for investment without evidence of return on investment or net operational value.

Regulatory and ethical governance must also address auditability, accountability, privacy, model updates, data ownership, workforce surveillance, unequal access among processors, and human oversight. High-consequence decisions involving food safety, carcass disposition, regulatory inspection, or employment should retain appropriate human review, particularly when confidence is low or inputs differ from the model’s training data (Floridi et al., 2018; Jobin et al., 2019).

Future research should develop diverse datasets representing multiple plants, seasons, processing lines, genetic populations, product types, sensors, and defect-prevalence conditions. Standardized annotations, enrichment of rare defects, independent external test sets, and comparisons with trained inspectors and existing commercial systems are needed. Multimodal and data-efficient approaches, including transfer, self-supervised, semisupervised, active, and federated learning may improve performance and reduce annotation barriers, but their added complexity should be justified through external validation and economic benefit (Hüllermeier and Waegeman, 2021).

Ultimately, poultry-processing AI should be evaluated by its effect on the production system rather than predictive performance alone. Future studies should demonstrate that AI supports a defined operational decision, improves safety, quality, yield, sustainability, or working conditions, remains reliable over time, and generates economic value without introducing unacceptable technical, regulatory, ethical, or social risks.

## Conclusions

AI has considerable potential to improve poultry processing by converting images, spectra, sensor measurements, and production records into timely and actionable information. Across the processing chain, AI-supported systems have demonstrated promising capabilities in carcass inspection, contamination and foreign-material detection, anatomical localization, breast-muscle abnormality classification, meat-quality and freshness prediction, microbial-risk assessment, yield estimation, robotic cutting, process monitoring, and package inspection. These applications could improve inspection consistency, product recovery, food-safety control, resource efficiency, worker safety, and responsiveness to the biological variability inherent in poultry products. Nevertheless, the maturity of the evidence differs substantially among applications. Commercial-line spectral carcass inspection and selected online quality-assessment systems provide stronger evidence of industrial relevance, whereas many deep-learning, robotic, packaging, and autonomous-control applications remain at the laboratory or pilot stage.

The central limitation of current research is the frequent gap between high model performance under controlled conditions and reliable operation in commercial plants. Restricted datasets, uncertain reference standards, class imbalance, data leakage, limited external validation, and inadequate reporting of uncertainty and class-specific errors can produce overly optimistic conclusions. Moreover, model accuracy alone does not establish operational value. An industrial system must acquire data consistently, generate predictions at line speed, tolerate sanitation and environmental variation, identify unfamiliar inputs, communicate uncertainty, and initiate an appropriate intervention without creating unacceptable product loss or food-safety risk. Performance should therefore be evaluated through processing outcomes including yield, false rejection, microbial control, product damage, throughput, downtime, resource consumption, and economic return rather than predictive metrics alone.

Future development should prioritize representative multi-flock, multi-season, and multi-plant datasets; biologically appropriate data partitioning; prospective commercial validation; standardized reporting; calibrated uncertainty; and out-of-distribution detection. AI systems must also be integrated with hygienically designed sensors, edge computing, existing plant equipment, traceability platforms, and validated control procedures. Human oversight will remain essential, particularly for uncertain predictions and high-consequence decisions involving food safety, regulatory inspection, and product disposition. Ultimately, the pathway toward intelligent and autonomous poultry processing will depend not on developing increasingly complex algorithms, but on demonstrating that integrated AI systems remain accurate, safe, explainable, economically justified, and operationally beneficial under realistic and changing commercial conditions.

## Author Contribution

**Zahidul Hasan Tushar:** Writing – original draft, Investigation, Formal analysis, Data curation, Methodology, Conceptualization. **Md. Salman Mostafa:** Writing – review & editing, Data curation.

## Disclosures

The authors declare that they have no known competing financial interests or personal relationships that could have appeared to influence the work reported in this paper.

## Footnotes

Supplementary material associated with this article can be found, in the online version, at doi:10.1016/j.psj.2026.107734.
