# Supplementary material for: Artificial intelligence in poultry processing: applications, validation gaps, and pathways toward intelligent and autonomous processing systems
Source: Poult Sci. 2026 Sep 7;105(12):107734. doi: 10.1016/j.psj.2026.107734 (PMC13594828; doi:10.1016/j.psj.2026.107734)
Supplement: Supplementary file 1 [file mmc1.docx]

**Supplementary Table S1. Detailed evidence map of artificial-intelligence and data-driven applications relevant to poultry processing**

| **Study** | **Task** | **Modality / data** | **AI / analytical approach** | **Sample size / plant context** | **Validation type** | **Reported performance** | **Deployment status / critical interpretation** |
| --- | --- | --- | --- | --- | --- | --- | --- |
| **Mortensen et al., 2016** | Live-bird weight estimation (preharvest/receiving-linked) | Kinect depth camera; 3D surface measurements | Bayesian artificial neural-network regression | ~48,000 broilers housed commercially; measurements obtained during grow-out rather than at plant receiving | 70/30 train/test split (189/83 broilers). 10‑fold CV for model selection. | Bayesian ANN: Mean relative error = 7.8% (σ=6.6%) across all ages. Daily mean error = 4.5% (avg absolute 55 g). Absolute errors: 20–100 g (early) → 50–250 g (late). All 5 regression models performed similarly. | Commercial-context (preharvest). Useful for anticipating processing weight variation, but should not be described as receiving-line deployment. |
| **Pirompud et al., 2024** | Prediction of high dead-on-arrival (DOA) in broilers | Rearing, catching, transport, body-weight, stocking-density, and timing records | LASSO, classification tree, random forest + imbalance-sampling methods | 22,115 truckloads from a large Thai producer (2021–2022); ante-mortem DOA recorded at slaughterhouse | 70:30 train/test split with 10-fold CV on training data | Original data: RF best (AUC=0.73 test, Se=0.06). With RUS balancing: RF (AUC=0.73, Se=0.67, F1=0.52); LASSO (AUC=0.64, Se=0.64); CT (AUC=0.59, Se=0.65). RUS significantly improved sensitivity vs. original. Top predictors: mortality/culling rate, rearing stocking density, season, mean body weight. | Commercial-context / retrospective. No prospective decision-support deployment was demonstrated. |
| **Jainonthee et al., 2025a** | Prediction of high DOA in meat-type ducks | Truckload, farm, transport, lairage, age, body weight and temperature records | LASSO, SVM, decision tree, random forest, XGBoost with resampling | 18,643 truckloads from 45 farms; 23,191,809 ducks delivered to one slaughterhouse (2019–2023) | Internal model development with cross-validation plus held-out test set | Original XGBoost test AUC 0.765 and sensitivity 0.334; best resampled model (XGBoost-Up) AUC 0.769, sensitivity 0.642, precision 0.476, F1 0.546 | Commercial-context / retrospective. Single slaughterhouse; no prospective deployment. |
| **Jainonthee et al., 2025b** | Explainable deep-learning prediction of high DOA in meat-type ducks | Preslaughter management and environmental truckload records | Deep neural network + SHAP | 8,220 duck truckload entries recorded during 2022–2023 | Internal train/test evaluation | Accuracy 80.29%; precision 79.25%; recall 80.29%; F1 79.66%; AUC-ROC 76.03% | Commercial-context / retrospective. Explainability added, but external slaughterhouse validation was not reported. |
| **Ye et al., 2018** | Electrical-stunning level classification | RGB/computer-vision images (320×240) | Back-propagation neural network using image-derived area/region features | 300 images; reported test group n=61 | Internal development/test split | Overall test accuracy 90.11% | Laboratory/pilot-line evidence. Feature-engineered model; no independent plant validation. |
| **Ye et al., 2020a** | Broiler stunned-state detection | RGB images collected from slaughter-line conditions | Improved convolutional neural network / YOLO-based residual approach | Line-derived image dataset; exact independent-plant sample size not reported in the source excerpt reviewed | Internal image-dataset validation | Reported accuracy 96.77%; study reported computational capacity >180,000 broilers/h | Pilot/line-derived. Reported computational speed is not equivalent to validated end-to-end plant throughput. |
| **Ye et al., 2020b** | Broiler stunned-state detection | RGB/CMOS slaughter-line images | Faster R-CNN + multilayer residual module | 2,319 original images augmented to 27,828; test set n=5,568 in the published study | Internal train/validation/test image split after augmentation | Accuracy 98.06%; class sensitivities approximately 98.41%, 98.20% and 97.31%; reported >40,000 birds/h inspection capacity | Pilot/line-derived. Single-image-domain validation; external plant testing not reported. |
| **Park et al., 2002** | Fecal and ingesta contamination detection | Hyperspectral imaging, 400–900 nm | PCA-guided wavelength selection and band-ratio image processing | Contaminated poultry carcasses; exact carcass n not stated in the accessible abstract reviewed | Controlled carcass experiment | Detection accuracy 97.3% with linear histogram stretching and 100% with nonlinear histogram stretching | Laboratory/pilot. Demonstrated technical feasibility; not a prospective high-speed commercial-line validation. |
| **Park et al., 2006** | Fecal and ingesta contamination detection | Hyperspectral imaging (430–900 nm, 512 bands; SensiCam CCD, ImSpector V9 spectrograph) | Single‑term linear regression (STLR) for wavelength selection. Band‑ratio classifiers: 2‑band (I565/I517) and 3‑band [(I576−I616)/(I529−I616)]. Threshold‑based binary classification. | 64 broiler carcasses | 1,066 manually applied contaminants; ground truth ROI spectra. Duncan's multiple range test (n=64). | 96.2% overall prediction accuracy; two-wavelength ratio 96.4% with 147 false positives | Laboratory/pilot. False positives remained important for practical inspection. |
| **Heitschmidt et al., 2007** | Fecal and ingesta contamination detection | Hyperspectral imaging, ~400–1000 nm | Decision-tree / selected-wavelength classification | 56 stationary carcasses; 1,030 applied fecal/ingesta contaminants | 1030 contaminants manually applied; results compared vs. 565/517 nm ratio alone. | >99% of contaminants identified; 10 false negatives and 25 false-positive pixels across 16 birds | Laboratory/pilot. Improved specificity relative to earlier versions, but stationary carcasses are not equivalent to line-speed operation. |
| **Lai and Kuo, 2023** | Carcass-part localization and defect classification | RGB machine vision | YOLOv7 localization followed by region-specific classification | Native-chicken carcass image dataset; single study environment | Internal image-dataset validation | Body-part detection mAP 98.9%; defect-classification accuracy 94.2% | Pilot/laboratory. Strong internal image metrics; multi-plant validation not demonstrated. |
| **Tran et al., 2024** | Carcass defect detection, segmentation and classification | RGB imaging from a university pilot processing plant | CarcassFormer transformer architecture | 7,321 images (4,279 single-carcass; 3,042 multiple-carcass); test set n=900 | Internal train/validation/test split; single pilot plant | For multiple-carcass images with ResNet-50 backbone: detection AP 90.45, defect AP 87.49, segmentation AP 98.96 | Pilot. Rich multi-task output, but no external-plant or full-shift prospective validation. |
| **Hasan et al., 2025** | Automated poultry quality inspection and sorting | RGB/computer-vision imaging | YOLOv8 object detection (trained 150 epochs) | Lab‑scale prototype | Manual inspection comparison across 5 trials (varied sample sizes: 25–45 chickens/trial). Performance metrics: precision, recall, mAP, confusion matrix. | System accuracy: Quality chickens – 95.33% (range 91.4–100%); Defect detection – 81.35% (range 71.4–100%). Precision: Chicken 98.4%, Defect 83.1%; Recall: Chicken 96.6%, Defect 73.5%. mAP@0.5 >90%, mAP@0.5:0.95 = 62%. | Research prototype. Demonstrates feasibility of low‑cost smartphone‑based automated sorting. |
| **Chen and Wang, 2018** | Visceral contour localization for evisceration | RGB machine vision; HSV/LAB color segmentation | Active-contour and threshold segmentation | 30 Three-Yellow chickens + 30 Cherry Valley ducks | Controlled experimental comparison across two poultry types | Whole-viscera recognition 93.3% in chickens and 86.7% in ducks | Laboratory/pilot. Performance differed by poultry type, illustrating domain-transfer limitations. |
| **Chen et al., 2020** | Robotic evisceration positioning | RGB machine vision + robotic manipulator/end effector | Image localization integrated with evisceration prototype | Experimental robotic system; two carcass-weight ranges | Prototype system evaluation | Maximum localization deviation 5 pixels (carcass) and 4 pixels (incision); image processing 954 ms; viscera residue 6.2%; breakage 15% | Pilot/prototype. Operational outcomes were substantially less favorable than image-localization metrics. |
| **Chen et al., 2021** | Poultry viscera segmentation/positioning | RGB machine vision | Improved region-based active contour / level-set segmentation | 100 visceral images of Three-Yellow chickens and Cherry Valley ducks used for algorithm comparison; equal numbers of chickens and ducks tested | Controlled algorithm comparison | Overall viscera segmentation accuracy 98.96%; heart-liver area 98.98%; fat area 99.75% | Laboratory/pilot. Pixel segmentation accuracy does not itself measure organ damage or residue. |
| **Zhu et al., 2025** | Prediction of internal visceral dimensions | 3D point-cloud imaging | GA‑WNN (Genetic Algorithm‑optimized WNN); inputs: carcass mass/length/width (3 nodes); outputs: visceral length/depth/upper left liver position (3 nodes); 6 hidden nodes, Morlet wavelet, 5‑fold CV; compared vs. RF, WNN, BP, GA‑BP, CNN. | Lab‑scale (Wuhan, China). 100 Sanhuang chickens. Generalization: 20 new Sanhuang + 100 black‑boned chickens + 100 partridge ducks. | 5‑fold cross‑validation (K=5). Test set 20% per fold. MAPE and RMSE for evaluation. | GA‑WNN best overall: Upper left liver: MAPE 3.47%, RMSE 0.508 cm; Visceral length: 3.22%, 0.589 cm; Visceral depth: 2.13%, 0.083 cm. Outperformed RF, WNN, BP, GA‑BP, CNN. Generalization: MAPE 2.3–4.3%, RMSE 0.087–0.606 cm across species. | Laboratory. Valuable external-domain check across breeds, but not integrated evisceration-line validation. |
| **Bolinger et al., 2021** | Post-chill *Salmonella*-risk prediction from microbiota | 16S rRNA microbiome profiles + culture-confirmed Salmonella status | Random forest and other ML models | 299 poultry rinsates: 82 prechill and 217 postchill; postchill model emphasized | 80/20 train/test split (post‑chill only); 3‑fold CV on training (n=174). | Best random forest: accuracy 88%, sensitivity 85%, specificity 90% | Laboratory/commercial-sample context. Prototype risk model, not a replacement for diagnostic testing. |
| **Yang et al., 2006** | Chilling/cross-contamination modeling | IBM tracking individual carcasses (N=80,000, 8‑h shift, 180 carcasses/min, 45‑min dwell). Variables: pathogen load/carcass (10⁴–10⁴.⁶ CFU) | Probability/mechanistic modeling | Lab‑scale (University of Arkansas). Data from simulated processing conditions, not industrial plant. | MSE and observed vs. predicted plots. | Weibull‑based secondary model selected (fewer parameters, MSEs <0.4). Predictions: C. jejuni more sensitive to heat/chlorine than S. Typhimurium. Skin protected bacteria vs. water. 8‑h chilling water had less effective chlorine than 0‑h water. | Research stage; not deployed. Models need validation under commercial processing conditions |
| **Yang et al., 2025** | Adaptive chicken deboning | Force sensing + 6-DoF robotic cutting | Residual reinforcement-learning policy with sim-to-real transfer | Simulation, physical multi-material testbed and real chicken shoulder demonstrations | Simulation + bench-scale + real-tissue proof of concept | Simulation: Adaptive success 0.80 (vs Nominal 0.20, w/o force 0.54); violation 4.74% (vs 18.1%, 9.04%). Testbed: Adaptive success 0.75, bone mass 0.065g (vs Nominal 0.05/3.032g; w/o force 0.35/0.730g). Real chicken: Adaptive success 0.50, bone mass 0.121g (vs Nominal 0.10/0.339g); 70% cuts had fragments <0.05g (vs 20%). | Laboratory/prototype; preprint/early evidence. Not a commercial-line study. |
| **Şahin et al., 2025** | Chicken-part classification for packaging | RGB images | YOLOv8 variants and RT-DETR | 907 images; 634 train, 136 validation, 137 test; five chicken-part classes | Random internal 70/15/15 split | Best YOLOv8s: F1 0.9969, mAP@0.5 0.9950, mAP@0.5:0.95 0.9807; 10.3 ms/image | Laboratory/packaging-context. High internal performance; no independent production-line test. |
| **Wold et al., 2017** | Wooden-breast detection and grading | Industrial NIR spectroscopy | LDA / protein-prediction-based discrimination | Calibration n=197; industrial test n=79; online scanning of three commercial flocks (9,063; 6,330; 10,483 fillets) | Independent industrial test + online commercial-line scanning | 99.5–100% calibration classification; 100% independent test classification | Commercial-line. Strong deployment evidence, but independent test set was small and multi-plant validation was not reported. |
| **Cai and Lu, 2025** | Wooden-breast detection | Light-scattering imaging | RLDA classifier | Lab‑scale (Michigan State University). 168 samples from commercial poultry plant (Orland, IN). | 10‑fold cross‑validation. | Deep features: 80.4%. Hand‑crafted full set: 91.7% (significantly better, P<0.01). MRMR top 65 features: 92.3%. PCA first 25 PCs: 92.3%. Confusion: Normal 88.9%, Defective 94.8% (MRMR). Scattering profiles: Defective samples attenuated light faster. | Laboratory. Useful evidence that more complex deep features are not automatically superior. |
| **Ekramirad et al., 2024** | Wooden-breast assessment | Optical coherence tomography (930 nm, 5 mm B‑scans, 2028 A‑scans/B‑scan, 2.44 µm lateral, 3.31 µm depth resolution) | SVM, kNN, LDA, NB, DT, RF, XGBoost. | Lab‑scale (USDA‑ARS, Athens, GA). 30 fillets from commercial plant, scanned ~3 h postmortem. | Repeated holdout (80/20 train/test, 5 iterations). PCA for visualization. | Normal vs. severe: 100% (RF, DT, SVM). Normal vs. all WB: 95% (DT, LDA). Normal vs. moderate: 95% (DT, LDA). Moderate vs. severe: 75% (DT, RF). Three‑class (normal/moderate/severe): best 86.6% (RF). Epimysium thickness clearly higher in WB. | Laboratory/feasibility. No commercial-line validation. |
| **Li et al., 2026** | Wooden-breast / quality sensing | Ultrasound imaging (7.5 MHz probe) of chicken breasts (Arbor Acres, 42 days, n=240) | Three CNNs: MobileNetV3, ResNet18, AlexNet. | Lab‑scale. 240 samples | 80/20 train/test split + 5‑fold cross‑validation. | MobileNetV3: training accuracy 99.50%, testing 98.71% (NORM 95%, MILD 100%, MOD 100%, SEV 99%). Precision 98.25%, recall 98.22%, F1 98.23%. ResNet18: 96.62%/90.09%; AlexNet: 95.64%/92.95%. Stratification: MILD ~55%, MOD ~62%, SEV ~65% of tissue affected. | Research/prototype stage. Ultrasound + DL enables non‑destructive, low‑cost, portable WB assessment with internal spatial information |
| **Ghorbanizamani, 2025** | Chicken spoilage / histamine monitoring | Color-shifting silver nanoparticles + smartphone RGB imaging | Artificial neural network | Real chicken-meat samples monitored over three days; analytical sensor calibration also performed | 70/15/15 train/validation/test split. Repeatability (CV% <10%) and reproducibility (CV%=1.93%) assessed. | ANN lowered histamine LOD to 0.09 μg/mL and extended dynamic range to 0.5–200 μg/mL | Laboratory. This is a quantitative sensor study |
| **Ellis et al., 2002** | Microbial spoilage quantification | ATR-FTIR spectroscopy on chicken meat | PLS regression + genetic programming | 25 comminuted 30-g chicken-breast samples per experiment; hourly measurements during 24 h spoilage; three spoilage experiments | Controlled repeated spoilage experiments | Bacterial loads predicted from FTIR in ~60 s; study emphasized quantitative agreement with plate counts rather than a classification accuracy | Laboratory. Early proof of concept; comminuted/ambient-spoilage conditions limit direct commercial transfer. |
| **Siddique et al., 2024** | Chicken breast spoilage-stage classification | NIR spectroscopy (350–2500 nm) | BPNN (250,000 steps, LR=0.02, 5 hidden layers) and SVM‑Linear for classification. Also regression models (PLS, SVR, GBR, MLP, RF). Feature extraction via bootstrap forest + variable clustering. | Lab‑scale (Auburn University) with commercial tray‑packs. Not industrial. | 10‑fold cross‑validation with nested 5‑fold CV for hyperparameter tuning. | BPNN classification: Initiation 93.7%, Propagation 95.2%, Spoiled 98%. SVM: overall 75.9%. Regression: best RF (MSE=0.17, R²=0.936) and GBR (R²=0.954). Extracted 10 signature wavelengths: 385, 400, 432, 1141, 1321, 1374, 2241, 2292, 2311, 2412 nm. | Laboratory. Controlled storage conditions may encode storage-time patterns. |
| **Spyrelli et al., 2020** | Chicken microbial quality / spoilage prediction | Spectroscopic/imaging data | PLS‑R (Partial Least Squares Regression). Pre‑processing: SNV or baseline offset; log transformation of target variable. | Industrial‑scale: Greek poultry plant. n=104 (breast), 97 (thigh), 131 (burger), 144 (souvlaki). Multiple batches. | Internal/controlled validation | Best: marinated souvlaki (r=0.934, RMSE=0.348); breast (r=0.886, RMSE=0.383); thigh (r=0.859, RMSE=0.160); burger (r=0.778, RMSE=0.285). | At‑line prototype, not fully deployed. |
| **Spyrelli et al., 2021a** | Microbial load and spoilage classification | Multispectral imaging of chicken thighs | PLS/SVM-type regression and SVM classification | 402 chicken-thigh fillets; controlled storage with independent-batch assessment | Internal modeling plus independent-batch validation | RMSE ~0.99 log CFU/cm² for TVC and 1.22 log CFU/cm² for Pseudomonas; fresh/spoiled SVM accuracy 94.4%; independent-batch errors were larger | Laboratory with stronger batch validation. Useful example of performance degradation outside calibration data. |
| **Arning et al., 2021** | *Campylobacter* source attribution | Whole-genome sequencing data | Machine learning | Campylobacter genomic datasets from multiple potential sources; not a direct poultry-processing-line study | Phylogeny‑aware train/test split (75/25) by ST; 5‑fold CV; 200 rounds of resampling with replacement. | Best (XGBoost + cgMLST): balanced accuracy 85%, imbalanced 84.6%. MLST: 71%, WGS k‑mers: 78%. iSource MLST: 64%. Fast prediction (892 ms for 15,988 human samples). | Laboratory/data-analytic. Relevant to food-safety attribution, not inline detection. |
| **Benefo et al., 2024** | Identification of Salmonella stress-response genes | Genomic/transcriptomic isolate features | Six supervised ML algorithms | Salmonella isolates associated with poultry processing | Train/test split (70/30), 10‑fold cross‑validation, evaluated via AUROC, sensitivity, specificity, Cohen’s κ. | Best: LB (AUROC=0.904, sensitivity=0.889, specificity=0.920, κ=0.81). Others: RF 0.855, CSL 0.872, SVML 0.895, etc. Identified top 20 genes (e.g., ybtX, rpoH, rpoE, cspA, fyuA, etc.) related to heat/cold shock, transport, LPS, biofilm. | Laboratory/data-analytic. Mechanistic insight rather than inline food-safety control. |
| **Park et al., 2023** | Bacterial-cell detection/segmentation in poultry rinsate | Hyperspectral microscopic imaging | Four U‑Net variants (U‑Net, ResU‑Net, AGResU‑Net, AGR2U‑Net) | Lab‑scale (USDA‑ARS), not industrial. 71 labeled hypercubes for training | Test set MIOU; manual ground‑truth masks (expert, ~19 min/image); EF results vs. expert‑labeled 6,426 blobs. | Best AGR2U‑Net: MIOU = 94.1%. EF accuracy = 97.4% (sensitivity 99.6%, specificity 88.4%). Segmentation ~15 sec/hypercube | Laboratory. Microscopic sample analysis, not direct carcass-line pathogen detection. |
| **Barbin et al., 2015** | Chicken meat quality prediction | NIR spectroscopy, 400–2500 nm | PCA + PLS regression | 158 breast fillets from two processing-plant batches | Cross-validation within two-batch dataset | L*: R²CV 0.91, SECV 1.99; pH: R²CV 0.81, SECV 0.07; WHC: R²CV 0.70, SECV 2.40% | Laboratory/commercial-sample context. Cross-validation is not equivalent to external plant validation. |
| **Barbon et al., 2018** | Chicken quality-grade classification | NIR spectra (1,050 wavelengths) + pH/color attributes | Decision trees / REPTree / SVM with wavelength selection | 158 samples: PSE n=24, pale n=86, normal n=41, DFD n=7 | Repeated resampling/internal classification | Best reduced-wavelength REPTree precision 77.2%; class imbalance was substantial | Laboratory. Important counterexample to headline >90% performance; class imbalance and minority classes matter. |
| **Kamruzzaman et al., 2016** | Chicken adulteration in minced beef | Hyperspectral imaging, 400–1000 nm | PLS-regression variants + pixel-level image prediction | 132 Samples,  Lab‑scale | Controlled calibration/prediction experiment | Full spectra: LS‑SVM best (R²p: L=0.96, a=0.95, b=0.85; RMSEP: 1.57, 1.12, 1.09; RPD: 4.95, 4.73, 2.88). With 6‑wavelength MLR: R²p=0.97 (L), 0.84 (a), 0.82 (b); RMSEP=1.72, 1.73, 1.35; RPD=4.52, 3.06, 2.33. | Laboratory. Beef-focused adulteration study with chicken as adulterant, not poultry-processing-line validation. |
| **Parastar et al., 2020** | Meat adulteration / authenticity | Handheld NIR (908–1676 nm, 125 vars), 3 measurement modes (on meat, through top, through bottom). 153 fresh + 133 thawed, 4,590 spectra total. | RSDE, PLS-DA, ANN, SVM | Total 153, Retail/lab‑scale (Dutch/Irish suppliers) | Train/test (70/30), leave‑chicken‑out CV, leave‑class‑out, y‑randomization. | Fresh/thawed: best test Acc = 92% (TP). Growth conditions: RSDE = 79–85% (single modes), 99.4% when combining 3 modes. Outperforms SVM (82%), CP‑ANN (65%), PLS‑DA (63%). | Laboratory; multi-species food-authenticity evidence. |
| **Fengou et al., 2021** | Pork–chicken adulteration detection | Multispectral imaging | SVM classification | Fresh n=360; stored n=180 at 24/48 h; cooked n=180; samples sourced from 12 butcher shops | External validation sets for fresh, stored and cooked conditions | Accuracy: fresh 96.67%; stored 93.33% (0 h), 97.78% (24 h), 95.56% (48 h); cooked 95.56% | Laboratory with external-condition validation. Stronger than random internal splitting, but not a commercial-line study. |
| **Pan et al., 2022** | Package-seal / package-integrity inspection | High-resolution RGB or line-scan imaging | Supervised autoencoder neural network | Food-packaging study | Study-specific validation | Average correlation coefficient: 0.6703 | General-food evidence |
| **Astuti et al., 2021** | Fresh vs *E. coli-*contaminated chicken classification | Electronic nose / six-gas-sensor array | Random forest and SVM | Controlled fresh and experimentally *E. coli*-contaminated chicken samples | Internal controlled classification | RF: 99.25% (fresh) and 98.42% (contaminated) precision/accuracy; SVM: 98.61% and 86.66% accuracy | Laboratory. No GC-MS confirmation of volatile composition; controlled contamination limits field generalization. |
| **Chen et al., 2016** | Total viable count prediction in chicken | Colorimetric sensor array / artificial olfaction | BP-AdaBoost regression | Chicken samples under controlled freshness/spoilage conditions | Prediction-set evaluation | RMSEP 0.543 log CFU/g; Rp 0.8119 | Laboratory. Regression error is more informative than freshness-classification accuracy. |
| **Ma et al., 2022** | Integrated freshness-indicator system | Colorimetric/fluorescent indicator with digital imaging | Pattern recognition / ML | Food-freshness indicator study | Study-specific validation | 97.1–100% pooled accuracy | Unknown/general-food evidence. |

Abbreviations: AUC/AUROC, area under the receiver-operating-characteristic curve; BP, back propagation; CNN, convolutional neural network; CV, cross-validation; DOA, dead on arrival; FTIR, Fourier-transform infrared; LASSO, least absolute shrinkage and selection operator; LDA, linear discriminant analysis; mAP, mean average precision; NIR, near-infrared; PCA, principal-component analysis; PLS, partial least squares; RF, random forest; RMSE/RMSEP, root mean square error/prediction; SHAP, Shapley additive explanations; SVM, support-vector machine; VIS/NIR, visible/near-infrared; WB, wooden breast; YOLO, You Only Look Once.

**REFERENCES**

Arning, N., S. K. Sheppard, S. Bayliss, D. A. Clifton, and D. J. Wilson. 2021. Machine learning to predict the source of campylobacteriosis using whole genome data. PLoS Genet. 17:e1009436. doi:10.1371/journal.pgen.1009436

Astuti, S. D., M. H. Tamimi, A. A. S. Pradhana, K. A. Alamsyah, H. Purnobasuki, M. Khasanah, Y. Susilo, K. Triyana, M. Kashif, and A. Syahrom. 2021. Gas sensor array to classify the chicken meat with *E. coli* contaminant by using random forest and support vector machine. Biosens. Bioelectron. X 9:100083. doi:10.1016/j.biosx.2021.100083

Barbin, D. F., C. M. Kaminishikawahara, A. L. Soares, I. Y. Mizubuti, M. Grespan, M. Shimokomaki, and E. Y. Hirooka. 2015. Prediction of chicken quality attributes by near infrared spectroscopy. Food Chem. 168:554–560. doi:10.1016/j.foodchem.2014.07.101

Barbon, S., A. P. A. Da Costa Barbon, R. G. Mantovani, and D. F. Barbin. 2018. Machine learning applied to near-infrared spectra for chicken meat classification. Journal of Spectroscopy. 2018:8949741. doi:10.1155/2018/8949741

Benefo, E. O., S. Karanth, and A. K. Pradhan. 2024. A machine learning approach to identifying Salmonella stress response genes in isolates from poultry processing. Food Research International 175:113635. doi:10.1016/j.foodres.2023.113635

Bolinger, H., D. Tran, K. Harary, G. C. Paoli, G. K. P. Guron, H. Namazi, and R. Khaksar. 2021. Utilizing the microbiota and machine learning algorithms to assess risk of *Salmonella* contamination in poultry rinsate. J. Food Prot. 84:1648–1657. doi:10.4315/JFP-20-367

Cai, J., and Y. Lu. 2025. Detection of woody breast condition in broiler breast fillets using light scattering imaging. Journal of the ASABE. 68:13–24. doi:10.13031/ja.16108

Chen, Q., W. Hu, J. Su, H. Li, Q. Ouyang, and J. Zhao. 2016. Nondestructively sensing of total viable count (TVC) in chicken using an artificial olfaction system based colorimetric sensor array. J. Food Eng. 168:259–266. doi:10.1016/j.jfoodeng.2015.08.003

Chen, Y., and S. C. Wang. 2018. Poultry carcass visceral contour recognition method using image processing. Journal of Applied Poultry Research. 27:316–324. doi:10.3382/japr/pfx073

Chen, Y., K. Feng, J. Lu, and Z. Hu. 2021. Machine vision on the positioning accuracy evaluation of poultry viscera in the automatic evisceration robot system. Int. J. Food Prop. 24:933–943. doi:10.1080/10942912.2021.1947315

Chen, Y., S. Wang, J. Bi, and H. Ai. 2020. Study on visual positioning and evaluation of automatic evisceration system of chicken. Food and Bioproducts Processing. 124:222–232. doi:10.1016/j.fbp.2020.08.017

Ekramirad, N., S. C. Yoon, B. C. Bowker, and H. Zhuang. 2024. Nondestructive assessment of woody breast myopathy in chicken fillets using optical coherence tomography imaging with machine learning: a feasibility study. Food and Bioprocess Technology. 17:4053–4070. doi:10.1007/s11947-024-03369-1

Ellis, D. I., D. Broadhurst, D. B. Kell, J. J. Rowland, and R. Goodacre. 2002. Rapid and quantitative detection of the microbial spoilage of meat by fourier transform infrared spectroscopy and machine learning. Appl. Environ. Microbiol. 68:2822–2828. doi:10.1128/aem.68.6.2822-2828.2002

Fengou, L. C., P. Tsakanikas, and G. J. E. Nychas. 2021. Rapid detection of minced pork and chicken adulteration in fresh, stored and cooked ground meat. Food Control. 125:108002. doi:10.1016/j.foodcont.2021.108002

Heitschmidt, G. W., B. Park, K. C. Lawrence, W. R. Windham, and D. P. Smith. 2007. Improved hyperspectral imaging system for fecal detection on poultry carcasses. Trans. ASABE 50:1427–1432. doi:10.13031/2013.23615

Jainonthee, C., P. Sanwisate, P. Sivapirunthep, C. Chaosap, D. Pichpol, R. Mektrirat, S. Chadsuthi, and V. Punyapornwithaya. 2025b. Predicting and explaining high dead-on-arrival outcomes in meat-type ducks using deep learning: A path to improved welfare management. Poult. Sci. 104:105439. doi:10.1016/j.psj.2025.105439

Jainonthee, C., P. Sanwisate, P. Sivapirunthep, C. Chaosap, R. Mektrirat, S. Chadsuthi, and V. Punyapornwithaya. 2025a. Data-driven insights into pre-slaughter mortality: Machine learning for predicting high dead on arrival in meat-type ducks. Poult. Sci. 104:104648. doi:10.1016/j.psj.2024.104648

Kamruzzaman, M., Y. Makino, and S. Oshita. 2016. Online monitoring of red meat color using hyperspectral imaging. Meat Sci. 116:110–117. doi:10.1016/j.meatsci.2016.02.004

Lai, P. C., and Y. F. Kuo. 2023. Detecting carcass defects of native chickens using convolutional neural networks. 2023 ASABE Annual International Meeting. doi:10.13031/aim.202301014

Li, L., Y. Ye, Z. Li, P. Wang, X. Xu, X. Sun, Y. Zhang, Q. Lv, Y. Yang, T. Huang, L. Wang, Z. Chen, and R. Yuan. 2026. Deep learning-based classification and internal region stratification of wooden breast in broiler by using ultrasound imaging. Poult. Sci. 105:106534. doi: 10.1016/j.psj.2026.106534

Ma, P., W. Xu, Z. Teng, Y. Luo, C. Gong, and Q. Wang. 2022. An integrated food freshness sensor array system augmented by a metal–organic framework mixed-matrix membrane and deep learning. ACS Sens. 7:1847–1854. doi:10.1021/acssensors.2c00255

Mortensen, A. K., P. Lisouski, and P. Ahrendt. 2016. Weight prediction of broiler chickens using 3D computer vision. Comput. Electron. Agric. 123:319–326. doi:10.1016/j.compag.2016.03.011

Pan, J., Z. Yang, S. H. K. Yap, X. Zhang, Z. Xu, Y. Li, Y. Luo, E. Zamburg, E. X. Liu, C. K. Tham, and A. V. Y. Thean. 2022. Non-destructive online seal integrity inspection utilizing autoencoder-based electrical capacitance tomography for product packaging assurance. Food Packag. Shelf Life. 33:100919. doi:10.1016/j.fpsl.2022.100919

Parastar, H., G. van Kollenburg, Y. Weesepoel, A. van den Doel, L. Buydens, and J. Jansen. 2020. Integration of handheld NIR and machine learning to “Measure & Monitor” chicken meat authenticity. Food Control. 112:107149. doi:10.1016/j.foodcont.2020.107149

Park, B., K. C. Lawrence, W. R. Windham, and D. P. Smith. 2006. Performance of hyperspectral imaging system for poultry surface fecal contaminant detection. J. Food Eng. 75:340–348. doi:10.1016/j.jfoodeng.2005.03.060

Park, B., K. C. Lawrence, W. R. Windham, and R. J. Buhr. 2002. Hyperspectral imaging for detecting fecal and ingesta contaminants on poultry carcasses. Transactions of the ASAE 45:2017. doi:10.13031/2013.11413

Park, B., T. Shin, R. Kang, A. Fong, B. McDonogh, and S. C. Yoon. 2023. Automated segmentation of foodborne bacteria from chicken rinse with hyperspectral microscope imaging and deep learning methods. Comput. Electron. Agric. 208:107802. doi:10.1016/j.compag.2023.107802

Pirompud, P., P. Sivapirunthep, V. Punyapornwithaya, and C. Chaosap. 2024. Application of machine learning algorithms to predict dead on arrival of broiler chickens raised without antibiotic program. Poult. Sci. 103:103504. doi:10.1016/j.psj.2024.103504

Şahin, D., O. Torkul, M. Şişci, D. D. Diren, R. Yılmaz, and A. Kibar. 2025. Real-Time classification of chicken parts in the packaging process using object detection models based on deep learning. Processes 13:1005. doi:10.3390/pr13041005

Siddique, A., C. B. Herron, B. Wu, K. S. S. Melendrez, L. J. G. Sabillon, L. J. Garner, M. Durstock, A. Sanz-Saez, and A. Morey. 2024. Development of predictive classification models and extraction of signature wavelengths for the identification of spoilage in chicken breast fillets during storage using near infrared spectroscopy. Food Bioproc. Tech. 18:933–941. doi:10.1007/s11947-024-03499-6

Spyrelli, E. D., A. I. Doulgeraki, A. A. Argyri, C. C. Tassou, E. Z. Panagou, and E. N. George-John. 2020. Implementation of multispectral imaging (MSI) for microbiological quality assessment of poultry products. microorganisms. 8:552. doi:10.3390/microorganisms8040552

Spyrelli, E. D., O. Ozcan, F. Mohareb, E. Z. Panagou, and G. J. E. Nychas. 2021a. Spoilage assessment of chicken breast fillets by means of fourier transform infrared spectroscopy and multispectral image analysis. Curr. Res. Food Sci. 4:121–131. doi:10.1016/j.crfs.2021.02.007

Tran, M., S. Truong, A. F. A. Fernandes, M. T. Kidd, and N. Le. 2024. CarcassFormer: an end-to-end transformer-based framework for simultaneous localization, segmentation and classification of poultry carcass defect. Poult. Sci. 103:103765. doi:10.1016/j.psj.2024.103765

Wold, J. P., E. Veiseth-Kent, V. Høst, and A. Løvland. 2017. Rapid on-line detection and grading of wooden breast myopathy in chicken fillets by near-infrared spectroscopy. PLoS One 12:e0173384. doi:10.1371/journal.pone.0173384

Yang, H., S. Wang, Y. Li, and M. G. Johnson. 2006. Predictive models for the survival/death of *Campylobacter jejuni* and *Salmonella Typhimurium* in poultry scalding and chilling. J. Food Sci. 67:1836–1843. doi:10.1111/j.1365-2621.2002.tb08731.x

Yang, Z., A.-P. Hu, and H. Ravichandar. 2025. Towards automated chicken deboning via learning-based dynamically-adaptive 6-DoF multi-material cutting. https://arxiv.org/pdf/2510.15376

Ye, C. wen, K. Yousaf, C. Qi, C. Liu, and K. jie Chen. 2020a. Broiler stunned state detection based on an improved fast region-based convolutional neural network algorithm. Poult. Sci. 99:637–646. doi:10.3382/ps/pez564

Ye, C. wen, Z. wei Yu, R. Kang, K. Yousaf, C. Qi, K. jie Chen, and Y. ping Huang. 2020b. An experimental study of stunned state detection for broiler chickens using an improved convolution neural network algorithm. Comput. Electron. Agric. 170:105284. doi:10.1016/j.compag.2020.105284

Ye, C., K. Yousaf, Y. Zhao, R. Kang, B. Pang, and K. Chen. 2018. Effectiveness of computer vision system and back propagation neural network in poultry stunning prediction. Int. Agric. Eng. J. 27:289–297. https://www.scopus.com/pages/publications/85064277848

Zhu, Z., Y. Chen, L. Cai, J. Yang, K. Wen, J. Bao, Z. Hu, and D. Fu. 2025. Predictive method for poultry carcass visceral dimensions using 3D point cloud and Genetic Algorithm-based wavelet neural network. Poult. Sci. 104:104516. doi:10.1016/j.psj.2024.104516
